# Pharmacologic Modulation of the PAR-2–ERK Axis by Statins Converts Inflammatory Survival Signalling into Apoptosis in Colorectal Cancer Cells

**DOI:** 10.3390/ijms27020916

**Published:** 2026-01-16

**Authors:** Layla Amiri, Rajashree Patnaik, Riah Lee Varghese, Bintul Huda, Yajnavalka Banerjee

**Affiliations:** Department of Basic Medical Sciences, College of Medicine, Mohammed Bin Rashid University of Medicine and Health Sciences, Dubai Health, Al Razi St, Umm Hurair 2, Dubai Healthcare City, Dubai 505055, United Arab Emiratesriah.varghese@dubaihealth.ae (R.L.V.);

**Keywords:** colorectal cancer, statins, PAR-2, ERK signalling, apoptosis, inflammation, atorvastatin, rosuvastatin, TNF-α, caspase activation

## Abstract

Chronic inflammation constitutes a well-established driver of colorectal carcinogenesis, yet the molecular circuitry linking inflammatory receptor signalling to tumour cell survival remains incompletely delineated. Here we demonstrate that the HMG-CoA reductase inhibitors atorvastatin and rosuvastatin modulate inflammatory survival pathways in colorectal cancer cells in a manner consistent with targeted interference with the protease-activated receptor 2 (PAR-2)–extracellular signal-regulated kinase (ERK)–tumour necrosis factor-α (TNF-α) signalling axis. Using lipopolysaccharide-stimulated HT-29 and Caco-2 cells as complementary models of inflammatory colorectal malignancy, we show that both statins selectively attenuate PAR-2 expression at the protein and transcript levels while leaving structurally related PAR-1 unaffected. This pattern of receptor modulation is accompanied by suppression of total ERK1/2 expression, ERK1/2 phosphorylation, and the transcriptional target DUSP6, together with attenuation of TNF-α secretion. Importantly, these signaling shifts are associated with dual apoptotic programs; the extrinsic pathway, reflected by transcriptional upregulation and proteolytic activation of caspase-8; and the intrinsic mitochondrial pathway, evidenced by reciprocal modulation of Bcl-2 family proteins favoring Bax over Bcl-2. Both pathways converge upon activation of executioner caspase-3 and an increase in Annexin V-defined apoptotic fractions, indicating re-engagement of programmed cell death under inflammatory stress. Notably, rosuvastatin consistently demonstrates superior potency across signaling endpoints, achieving comparable biological effects at lower concentrations than atorvastatin. Collectively, these data indicate that clinically deployed statins target the PAR-2–ERK axis and are associated with re-activation of apoptotic pathways in inflammatory colorectal cancer models, while leaving open the possibility that additional statin-responsive networks contribute to their pro-apoptotic effects. This mechanistic framework provides biological plausibility for epidemiologic observations linking statin use with reduced colorectal cancer risk and improved outcomes, and supports further translational evaluation of PAR-2-directed statin strategies in colorectal malignancy.

## 1. Introduction

Colorectal cancer (CRC) remains one of the most prevalent and lethal malignancies worldwide, imposing a substantial clinical burden. In addition to well-established genetic and environmental factors, epidemiological studies have increasingly implicated metabolic dysfunction in the pathogenesis of CRC [1,2]. Components of metabolic syndrome, including obesity, insulin resistance, and dyslipidemia, are associated with a higher incidence of colorectal neoplasia [3]. Elevated serum cholesterol and free fatty acids may create a pro-tumorigenic milieu by altering membrane composition, promoting oxidative stress, and modulating oncogenic signaling pathways in epithelial cells [4]. Consequently, strategies targeting lipid metabolism and related systemic factors are of growing interest for CRC prevention and therapy [5].

Statins, competitive inhibitors of 3-hydroxy-3-methylglutaryl-coenzyme A (HMG-CoA) reductase, are first-line agents for treating hyperlipidemia [6]. Beyond their effect on lowering cholesterol, statins exhibit pleiotropic anti-inflammatory and anti-neoplastic properties that are increasingly recognized in cancer research [7]. By blocking the mevalonate pathway, statins deplete isoprenoid intermediates such as farnesyl pyrophosphate and geranylgeranyl pyrophosphate, which are required for prenylation of proteins like RAS (Rat Sarcoma viral oncogene homolog) and RHO (Ras Homologous proteins) [8]. This impairs membrane localization and activity of these GTPases, resulting in attenuation of mitogenic signaling through the MAPK/ERK and PI3K/AKT pathways [9,10]. In CRC cell models, statins have been shown to induce cell-cycle arrest, inhibit proliferation, and trigger apoptosis, often accompanied by downregulation of the anti-apoptotic Bcl-2 and inhibition of NF–κB-regulated cytokine production [11,12]. Epidemiological evidence further suggests that regular statin use correlates with reduced colorectal adenoma and carcinoma risk, underscoring the potential for statins in CRC chemoprevention [13,14].

Chronic inflammation is a well-established driver of colorectal tumorigenesis [15]. Inflammatory bowel disease (IBD), for example, ulcerative colitis, dramatically increases the risk of CRC, linking persistent immune activation to neoplastic transformation [16]. At the cellular level, lipopolysaccharide (LPS), a component of Gram-negative bacterial cell walls, is commonly used to simulate an inflammatory microenvironment in CRC models [17]. LPS engages toll-like receptor 4 (TLR4) on colonic epithelial cells, activating downstream NF-κB and MAPK signaling pathways, which leads to the secretion of pro-inflammatory cytokines, such as TNF-α and IL-6 [18]. Notably, although TNF-α can induce apoptosis under certain conditions, chronic TNF-α signaling in the tumor microenvironment typically enhances tumor cell survival, proliferation, and angiogenesis via sustained NF-κB/ERK pathway activation [19].

Protease-activated receptor 2 (PAR-2) has emerged as a pivotal mediator linking extracellular inflammation to intracellular oncogenic signaling in CRC [20]. PAR-2 is a G-protein-coupled receptor activated by serine proteases (e.g., trypsin, tryptase) found in the tumor microenvironment, and its activation triggers G_q/11_ and G_i/o_ pathways leading to phospholipase C activation, Ca^2+^ mobilization, and ERK1/2 phosphorylation [21].

In CRC cells, PAR-2 stimulation enhances pro-tumorigenic processes, including proliferation, migration, and the secretion of additional inflammatory mediators [21]. Critically, PAR-2 signaling upregulates ERK1/2 phosphorylation and TNF-α expression while suppressing caspase-mediated apoptosis, thus establishing a feed-forward pro-survival loop [22]. By contrast, PAR-1, a related protease receptor, is not significantly affected by the same inflammatory stimuli in CRC [23,24]. In a previous proof-of-concept investigation, we demonstrated that atorvastatin and rosuvastatin selectively downregulate PAR-2 (but not PAR-1) in LPS-treated HT-29 and Caco-2 cells [25]. This specificity highlights PAR-2 as a prime target of statin action in the context of colorectal inflammation.

Building on these insights, the present study examines the downstream consequences of statin-mediated PAR-2 inhibition. We observed that treatment of LPS-stimulated CRC cells with atorvastatin or rosuvastatin significantly reduced TNF-α expression, as measured by ELISA and qRT-PCR [25]. Given that TNF-α transcription is driven by the PAR-2/ERK/NF-κB signaling axis [26,27], this finding suggests that statin treatment interrupts the inflammatory loop at an early stage. Consistent with this, Western blot analysis revealed that statin treatment markedly decreased the ratio of phosphorylated ERK1/2 to total ERK1/2 in the CRC cells [25], confirming suppression of MAPK pathway activation. Attenuation of ERK signaling likely curtails the transcription of pro-survival genes and dampens further cytokine secretion, reinforcing the shift away from a pro-inflammatory tumor phenotype [28].

Concomitantly, statin treatment shifted the balance toward apoptosis in the CRC cells. We detected increased cleavage (activation) of executioner caspase-3 and initiator caspase-8 in statin-treated cells, along with reductions in pro-caspase-8 and the anti-apoptotic protein Bcl-2, relative to LPS-only controls. These molecular effects were mirrored by functional assays: Annexin V/PI flow cytometry confirmed a higher percentage of apoptotic cells following statin exposure. Mechanistically, this profile is consistent with the statin blockade of NF-κB/ERK survival pathways, which frees caspase cascades to proceed and downregulates survival signals, such as Bcl-2. In effect, the chronic inflammatory stimulus is converted into a trigger for programmed cell death rather than unchecked proliferation.

This integrated mechanistic portrait underscores the novelty of our findings. To our knowledge, it is the first demonstration that clinically used statins can concurrently disable an inflammation-sensitive GPCR signaling axis and its downstream oncogenic effectors in CRC cells. By linking our previous observation of statin-induced PAR-2 downregulation [25] with the new evidence of reduced TNF-α, inhibited ERK1/2 activation, and enhanced apoptosis, we delineate a novel pathway by which statins convert pro-survival inflammatory signals into apoptosis. The multi-faceted nature of this blockade, targeting PAR-2 at the receptor level and multiple intracellular pathways, highlights the innovative aspect of this study.

We chose atorvastatin and rosuvastatin for their complementary pharmacological profiles. Atorvastatin is a lipophilic statin that readily crosses cell membranes and is extensively metabolized by hepatic CYP3A4 enzymes, whereas rosuvastatin is a highly potent hydrophilic statin with a longer half-life and minimal metabolic modification [29,30]. By testing both agents, we sought to determine whether lipophilicity or potency influences the anti-cancer effects [31,32,33]. Importantly, both statins elicited qualitatively similar outcomes in downregulating PAR-2 and inflammatory signaling [34,35], as well as promoting apoptosis, suggesting that these effects are likely a class effect of HMG-CoA reductase inhibition [36,37,38,39,40]. The dose-dependent responses observed at two concentrations of each statin further support the conclusion that the effects are pharmacologically specific and not due to off-target toxicity.

In summary, our study reveals that treatment with atorvastatin and rosuvastatin in an LPS-induced inflammatory CRC model disrupts tumor-promoting signaling at multiple levels, from receptor expression to cytokine production and apoptotic checkpoints. This multi-pronged mechanism highlights statins as promising agents to counteract inflammation-driven colorectal carcinogenesis [40,41,42,43]. Our findings not only corroborate and extend our previous observations, but also provide a detailed mechanistic rationale for repurposing statins in CRC management, particularly in patients with concomitant hyperlipidemia or chronic inflammation.

## 2. Results

### 2.1. Morphological Integrity and Lps-Induced Cytotoxicity Profiling in Ht-29 and Caco-2 Cells

Phase-contrast microscopy and metabolic viability analyses were performed to validate the structural fidelity and inflammatory responsiveness of HT-29 and Caco-2 cells following exposure to increasing concentrations of LPS. These experiments established the foundational inflammatory CRC model prior to statin intervention, ensuring that downstream molecular changes could be attributed to signaling perturbations rather than confounding cytotoxic effects.

**Figure 1:**
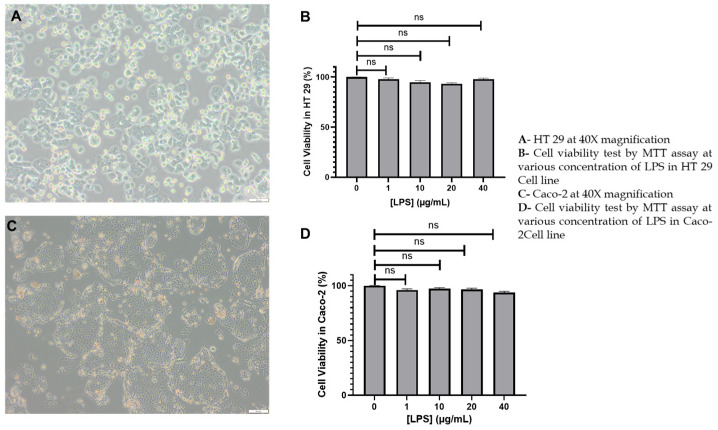
Morphological assessment and LPS-induced cytotoxicity profiling in HT-29 and Caco-2 colorectal cancer cell lines. (**A**) Phase-contrast micrograph of untreated HT-29 cells at 40× magnification demonstrating a proliferative epithelial monolayer with characteristic polygonal morphology, intact membranous boundaries, and preserved cytoplasmic integrity. The absence of blebbing, vacuolation, or detachment indicates optimal basal viability and confirms the structural readiness of HT-29 cells for downstream inflammatory stimulation; (**B**) MTT-based metabolic viability assessment of HT-29 cells exposed to escalating concentrations of LPS (1–40 µg/mL). Cell viability remained ≥90% across all doses, confirming that LPS engages inflammatory signaling without exerting nonspecific cytotoxic effects or impairing mitochondrial dehydrogenase activity; (**C**) Phase-contrast micrograph of untreated Caco-2 cells at 40× magnification displaying classical cobblestone epithelial architecture, dense apical–basolateral polarization, and uniform intercellular contacts. These features reflect preserved differentiation status and epithelial fidelity, validating the suitability of Caco-2 for modeling early-stage CRC inflammation; (**D**) MTT viability assay of Caco-2 cells treated with increasing concentrations of LPS (1–40 µg/mL). Viability consistently exceeded 95%, indicating preserved metabolic competence and confirming that the selected LPS dose (10 µg/mL) induces inflammatory activation without compromising cellular integrity. (Note: Together, these analyses establish the morphological stability and metabolic resilience of HT-29 and Caco-2 cells under LPS exposure, validating the use of 10 µg/mL LPS as an optimal non-cytotoxic dose for generating a reproducible in vitro inflammatory CRC model for subsequent mechanistic interrogation of statin-mediated signaling modulation. ns: refers to non-significant.)

At baseline, HT-29 cells imaged at 40× magnification (Figure 1A) exhibited the expected irregular, polygonal epithelial phenotype with moderate spreading and well-defined cell–cell interfaces. The cytoplasm displayed uniform granularity, and nuclei were centrally positioned with intact contours. No evidence of membrane blebbing, apoptotic shrinkage, or vacuolation was observed, confirming optimal viability under untreated conditions. The architecture reflected a proliferative monolayer with partial epithelial-mesenchymal plasticity, consistent with their established tumorigenic phenotype and suitability for studies on inflammatory signaling.

Caco-2 cells, likewise examined at 40× magnification (Figure 1C), formed tightly ordered epithelial sheets with a prototypical cobblestone morphology. Distinct apical–basolateral polarity was evident from differential refractive patterns, and intercellular junctions appeared continuous and compact, reflecting the high integrin and tight-junction expression characteristic of this line. Cytoplasmic homogeneity and absence of degenerative morphological cues further confirmed their suitability for modeling early-stage CRC epithelial barrier responses during inflammatory stimulation [44].

To assess whether LPS exposure produced nonspecific cytotoxicity that could confound downstream mechanistic analyses, both cell lines were subjected to escalating LPS concentrations (1, 10, 20, 40 µg/mL), and cell viability was quantified using the MTT assay. HT-29 cells (Figure 1B) demonstrated exceptional resilience, maintaining ≥90% viability across all concentrations. The absence of any significant decrement in formazan reduction implied that mitochondrial dehydrogenase activity remained intact and that LPS stimulation at these doses principally engaged signaling pathways rather than compromising metabolic homeostasis.

Caco-2 cells exhibited a similarly robust profile (Figure 1D), with viability consistently exceeding 95% across the entire range of LPS tested. No dose-dependent suppression of metabolic activity was detected, underscoring the capacity of Caco-2 to withstand LPS exposure without structural or metabolic compromise. The preservation of viability across concentrations confirms that LPS-TLR4 pathway activation occurs independently of mitochondrial dysregulation or cytotoxic stress.

Together, the morphological and metabolic assays demonstrate that both HT-29 and Caco-2 cells retain structural integrity and mitochondrial functionality after LPS exposure, thereby validating the use of 10 µg/mL LPS as an optimal concentration for inducing inflammation without introducing confounding toxicity. This ensures that any subsequent modulation of inflammatory mediators or downstream apoptotic signaling by atorvastatin or rosuvastatin can be attributed to pharmacological effects rather than perturbations of cell viability.

### 2.2. Atorvastatin and Rosuvastatin Attenuate Lps-Induced Par-2 Expression at the Protein and Transcript Levels in Ht-29 and Caco-2 Cells

**Figure 2:**
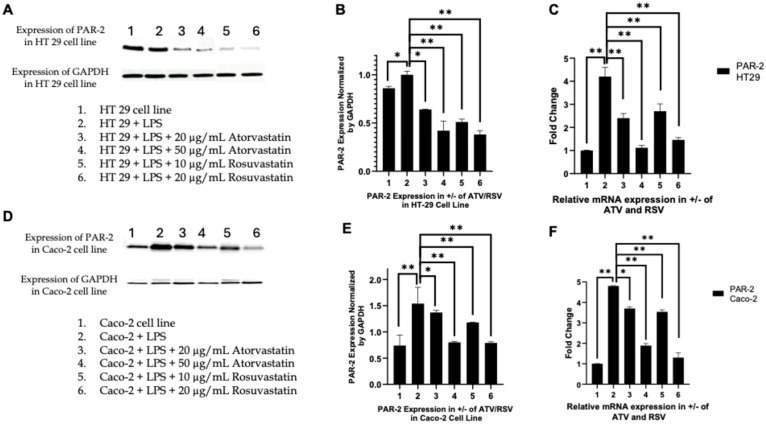
Atorvastatin (ATV) and Rosuvastatin (RSV) Attenuate LPS-Induced PAR-2 Expression at the Protein and Transcript Level in HT-29 and Caco-2 Colorectal Cancer Cells. (**A**) Representative immunoblots demonstrating the effect of LPS (10 µg/mL) and statin co-treatment on PAR-2 protein abundance in HT-29 cells. Lane assignments: 1-untreated control; 2-LPS only; 3-LPS + 20 µg/mL atorvastatin; 4-LPS + 50 µg/mL atorvastatin; 5-LPS + 10 µg/mL rosuvastatin; 6-LPS + 20 µg/mL rosuvastatin. GAPDH served as the loading control. LPS elicited a marked increase in PAR-2 expression relative to baseline, whereas co-treatment with either statin attenuated this induction in a concentration-dependent manner, with the highest suppression observed in the 20 µg/mL rosuvastatin condition; (**B**) Densitometric quantification of PAR-2 normalized to GAPDH confirms significant reductions across all statin-treated groups compared to LPS alone, with rosuvastatin demonstrating a comparatively greater magnitude of suppression than atorvastatin at equivalent doses (*p* < 0.05; *p* < 0.01); (**C**) Corresponding qPCR analysis shows parallel reductions in PAR-2 mRNA expression in HT-29 cells following statin treatment, mirroring the protein-level trends and indicating transcriptional downregulation (*p* < 0.01); (**D**) Immunoblots showing the expression profile of PAR-2 under identical treatment conditions in Caco-2 cells. LPS again induced robust PAR-2 upregulation, while statin co-treatment significantly mitigated this effect, with the strongest suppression observed following 20 µg/mL rosuvastatin; (**E**) Densitometric quantification corroborates a graded, dose-responsive decrease in PAR-2 protein levels, with rosuvastatin consistently producing greater inhibitory effects compared with atorvastatin (*p* < 0.05; *p* < 0.01); (**F**) qPCR analysis reveals significant attenuation of LPS-driven PAR-2 transcript induction, demonstrating concordance between transcriptional and translational responses to statin treatment in Caco-2 cells (*p* < 0.01). (Data are presented as the mean ± s.e.m. from three independent experiments. Statistical significance was determined by one-way ANOVA followed by Tukey’s post hoc test. * *p* < 0.05, ** *p* < 0.01.). (Note: Collectively, these findings establish that both atorvastatin and rosuvastatin counteract LPS-induced PAR-2 upregulation in CRC cell lines at the protein and mRNA levels, with rosuvastatin exhibiting a more pronounced suppressive effect across both cellular models.)

In HT-29 cells, Western blot analysis revealed that exposure to LPS significantly increased PAR-2 protein abundance compared to untreated controls, confirming a robust inflammatory response (Figure 2A). Treatment with atorvastatin resulted in a clear, concentration-dependent reduction in PAR-2 protein levels, with the higher dose producing a more pronounced attenuation. Rosuvastatin exerted a more pronounced suppressive effect; at equivalent or lower doses, PAR-2 immunoreactivity was diminished to a greater extent than that observed with atorvastatin. Densitometric quantification normalized to GAPDH (Figure 2B) corroborated these visual observations, showing statistically significant reductions in PAR-2 protein expression across all statin-treated groups, with rosuvastatin demonstrating the strongest overall suppression.

Transcriptomic analysis of PAR-2 mRNA in HT-29 cells (Figure 2C) revealed proportional trends. LPS stimulation significantly elevated PAR-2 transcription, whereas both statins reversed this induction in a dose-responsive manner. The decrease in mRNA expression closely paralleled the protein findings, with rosuvastatin again producing the most pronounced reduction. These data confirm that the statin-mediated attenuation of PAR-2 occurs at both transcriptional and translational readouts within the same cellular context.

A comparable pattern emerged in Caco-2 cells. LPS exposure significantly increased PAR-2 protein levels (Figure 2D), affirming that this line also mounts a reproducible PAR-2-dependent inflammatory response. Atorvastatin treatment reduced PAR-2 protein abundance in a dose-dependent manner, while rosuvastatin produced visibly deeper suppression, even at lower concentrations. Densitometric analyses (Figure 2E) supported these observations, revealing a significant decrease in PAR-2 protein across all statin-treated groups, with the highest dose of rosuvastatin producing the most pronounced decrement.

The transcript data in Caco-2 cells (Figure 2F) showed consistent directional alignment with the protein findings. PAR-2 mRNA was significantly elevated following LPS stimulation and was subsequently repressed by both statins. As in HT-29, rosuvastatin produced a quantitatively greater reduction in transcript levels when compared with atorvastatin at matched doses.

Taken together, these results demonstrate that both atorvastatin and rosuvastatin effectively counteract LPS-induced PAR-2 upregulation, reducing expression at the protein and mRNA levels across distinct colorectal cancer phenotypes. Although both agents exerted robust inhibitory activity [45], rosuvastatin consistently achieved greater suppression of PAR-2 expression in both HT-29 and Caco-2 models, suggesting a comparatively stronger regulatory impact in this inflammatory setting.

### 2.3. Statin Treatment Does Not Alter Par-1 Expression in Ht-29 or Caco-2 Cells, Confirming Specificity Toward Par-2

To establish whether the suppressive effects of atorvastatin and rosuvastatin on PAR-2 signalling extended to structurally related members of the PAR family, we examined PAR-1 expression as a mechanistically distinct comparator receptor. PAR-1 was selected based on its intermediate sequence identity with PAR-2, its autonomous G-protein-coupled receptor signaling capacity, and its well-characterized expression within colorectal epithelial systems, thereby providing an appropriate reference point for establishing the target selectivity of statin-mediated modulation of protease-activated receptor signaling. Unlike PAR-3, which functions predominantly as a cofactor in PAR-1/PAR-4 complexes, and PAR-4, whose colonic epithelial expression remains comparatively inconsistent, PAR-1 offers a physiologically relevant framework for interrogating the specificity of our observations [46].

**Figure 3:**
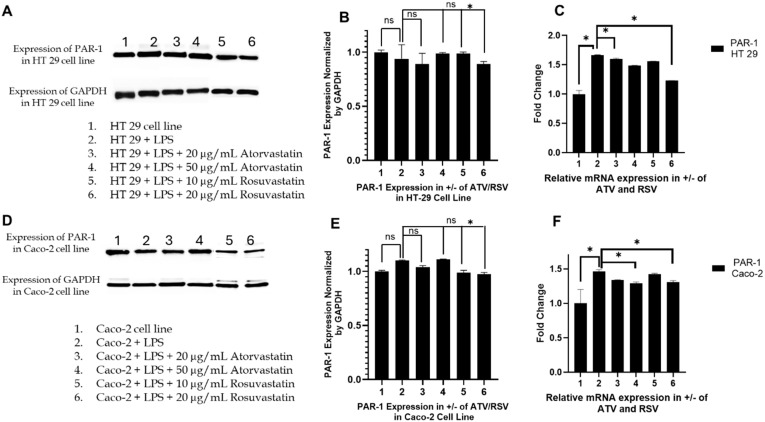
Atorvastatin (ATV) and Rosuvastatin (RSV Do Not Alter PAR-1 Expression in HT-29 or Caco-2 Cells, Demonstrating Selective Modulation of PAR-2 Signaling. (**A**) Representative Western blots depicting PAR-1 protein expression in HT-29 cells across untreated baseline, LPS stimulation (10 µg/mL), and statin co-treatment conditions. Lane assignments: 1—untreated control; 2—LPS only; 3—LPS + 20 µg/mL atorvastatin; 4—LPS + 50 µg/mL atorvastatin; 5—LPS + 10 µg/mL rosuvastatin; 6—LPS + 20 µg/mL rosuvastatin. GAPDH served as the loading control. PAR-1 protein levels remained stable across all conditions, with no detectable change in response to LPS priming or to either atorvastatin or rosuvastatin; (**B**) Densitometric quantification of PAR-1 normalized to GAPDH in HT-29 cells confirms the absence of significant differences among untreated, LPS-treated, and statin-treated groups (*p* > 0.05), indicating that PAR-1 is not modulated by inflammatory stimulation or by statin exposure; (**C**) qPCR analysis of PAR-1 mRNA expression in HT-29 cells demonstrates parallel transcriptional stability, with no significant LPS-induced induction and no measurable modulation by either concentration of atorvastatin or rosuvastatin (*p* > 0.05); (**D**) Representative immunoblots showing PAR-1 protein expression in Caco-2 cells under identical treatment conditions. As observed in HT-29 cells, neither LPS stimulation nor statin co-treatment produced visible alterations in PAR-1 band intensity relative to baseline. GAPDH remained consistent across all lanes; (**E**) Densitometric quantification of PAR-1 normalized to GAPDH in Caco-2 cells corroborates protein-level stability, with no statistically significant changes across treatment groups (*p* < 0.05); (**F**) Corresponding qPCR analysis reveals unchanged PAR-1 transcript levels in Caco-2 cells following LPS stimulation or statin co-treatment, mirroring the protein-level findings (*p* > 0.05). Data are presented as the mean ± s.e.m. from three independent experiments. Statistical significance was determined by one-way ANOVA followed by Tukey’s post hoc test. ns = non-significant, * *p* < 0.05. (Note: The complete absence of both transcriptional and translational modulation of PAR-1 across two distinct CRC cellular models underscores the target specificity of atorvastatin and rosuvastatin. Combined with Figure 2, these data demonstrate that statin-mediated regulation is selective for PAR-2 and does not extend to other protease-activated receptors.)

In HT-29 cells, Western blot analysis demonstrated that PAR-1 protein levels remained unaltered across all experimental conditions examined (Figure 3A). Basal PAR-1 expression in untreated cells was not altered by LPS priming. Subsequent co-treatment with atorvastatin at concentrations of 20 or 50 µg/mL, or rosuvastatin at 10 or 20 µg/mL, produced no discernible changes in immunoreactive band intensity. Densitometric quantification corroborated these visual observations, revealing no statistically significant deviation in PAR-1 protein abundance across any treatment group relative to vehicle-treated controls (Figure 3B). Uniform GAPDH expression across all lanes confirmed equivalent protein loading. Complementary transcriptomic analysis, utilizing quantitative polymerase chain reaction, revealed an analogous profile of transcriptional stability (Figure 3C). LPS stimulation elicited no significant induction of PAR-1 messenger RNA, and neither atorvastatin nor rosuvastatin produced measurable suppression or enhancement of transcript abundance. Fold-change values remained tightly clustered around unity across all treatment conditions, indicating pronounced transcriptional resistance of PAR-1 to statin exposure within the HT-29 cellular phenotype.

Caco-2 cells displayed a substantially equivalent response pattern at both molecular levels examined. Western blot analysis revealed uniform PAR-1 protein banding patterns across untreated, LPS-stimulated, and statin-treated cohorts, with no discernible attenuation or augmentation in response to either atorvastatin or rosuvastatin (Figure 3D). Densitometric quantification confirmed the absence of statistically significant variation relative to control conditions, reinforcing that statin treatment does not perturb PAR-1 protein abundance at the translational level (Figure 3E). Consistent with these protein-level observations, PAR-1 mRNA levels in Caco-2 cells remained unchanged across all experimental conditions (Figure 3F). Neither atorvastatin nor rosuvastatin altered transcriptional output, and LPS exposure alone was insufficient to induce PAR-1 expression, thereby demonstrating the relative transcriptional stability of this receptor under inflammatory conditions.

The complete absence of statin-mediated modulation of PAR-1 expression at both protein and mRNA levels, demonstrated across two distinct CRC cellular phenotypes, unequivocally establishes that the suppressive effects of statins on PAR signaling are specific to the PAR-2 axis. Whereas PAR-2 was robustly downregulated by both atorvastatin and rosuvastatin under identical experimental conditions, PAR-1 expression remained entirely refractory to statin exposure. These findings definitively establish that the observed molecular effects do not reflect a generalized suppression of the PAR receptor family, but rather constitute a targeted and selective regulatory action on PAR-2 signaling, thereby substantially strengthening the mechanistic specificity of our investigative conclusions.

### 2.4. Atorvastatin and Rosuvastatin Modulate Erk1/2 and Phosphorylated Erk Signaling in Ht 29 and Caco-2 Cells

Given that PAR-2 activation is known to engage downstream MAPK cascades that drive proliferative and inflammatory responses in colorectal cancer, we next investigated whether atorvastatin and rosuvastatin could attenuate ERK1/2 signalling, a principal effector arm of the PAR-2/MAPK axis. The ERK1/2 isoforms were selected for analysis based on their established roles in transducing mitogenic signals, sustaining inflammatory cytokine production, and promoting cell survival in CRC models following PAR-2 stimulation. We therefore assessed the expression of total ERK1/2 and their phosphorylated forms (p-ERK1/2) in HT 29 and Caco-2 cells treated with LPS alone or in combination with atorvastatin (20 µg/mL or 50 µg/mL) or rosuvastatin (10 µg/mL or 20 µg/mL).

**Figure 4:**
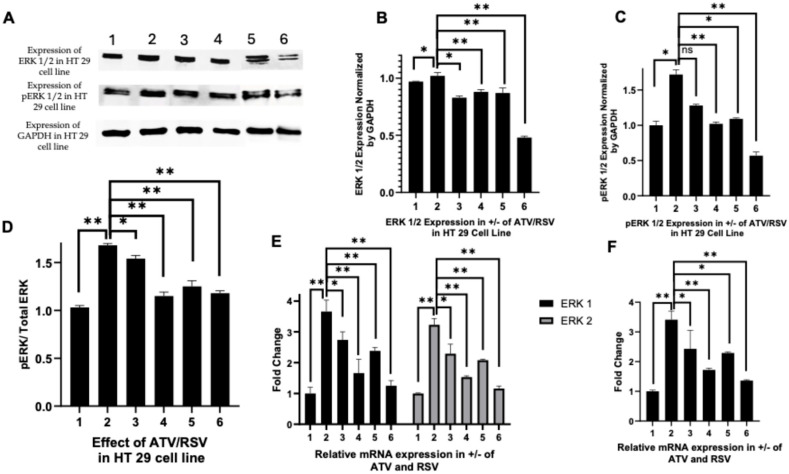
Atorvastatin and rosuvastatin suppress ERK1/2 signalling in LPS-stimulated HT 29 colorectal cancer cells. (**A**) Representative Western blot analysis of total ERK1/2, phosphorylated ERK1/2 (pERK1/2), and GAPDH (loading control) in HT 29 cells. Lanes represent: (1) untreated control; (2) LPS alone; (3) LPS + atorvastatin 20 µg/mL; (4) LPS + atorvastatin 50 µg/mL; (5) LPS + rosuvastatin 10 µg/mL; (6) LPS + rosuvastatin 20 µg/mL; (**B**) Densitometric quantification of total ERK1/2 expression normalised to GAPDH; (**C**) Densitometric quantification of pERK1/2 expression normalised to GAPDH; (**D**) Ratio of phosphorylated ERK to total ERK protein levels; (**E**) Quantitative RT-PCR analysis of ERK1 (MAPK3, black bars) and ERK2 (MAPK1, grey bars) mRNA expression relative to untreated controls; (**F**) Quantitative RT-PCR analysis of DUSP6 mRNA expression, a transcriptional target and negative feedback regulator of ERK signalling. (Data are presented as the mean ± s.e.m. from three independent experiments. Statistical significance was determined by one-way ANOVA followed by Tukey’s post hoc test. * *p* < 0.05, ** *p* < 0.01; ns, not significant.)

In HT 29 cells, Western blot analysis (Figure 4A) demonstrated that LPS stimulation (lane 2) induced a modest increase in total ERK1/2 protein levels compared to untreated controls (lane 1). Co-treatment with atorvastatin at 20 µg/mL (lane 3) and 50 µg/mL (lane 4) produced a progressive reduction in ERK1/2 expression, although this effect was relatively modest. Rosuvastatin treatment yielded more pronounced suppression, with the 20 µg/mL concentration (lane 6) reducing ERK1/2 levels to approximately half of those observed in LPS-stimulated cells. Densitometric quantification (Figure 4B) confirmed these observations, revealing statistically significant reductions in total ERK1/2 expression with both statins, with rosuvastatin at 20 µg/mL demonstrating the greatest suppressive effect (*p* < 0.01).

Analysis of phosphorylated ERK1/2 in HT 29 cells (Figure 4A, middle panel) revealed that LPS stimulation markedly elevated p-ERK levels (lane 2) compared to baseline (lane 1). Atorvastatin at 20 µg/mL (lane 3) did not significantly reduce p-ERK expression relative to LPS alone, as indicated by the non-significant comparison in the densitometric analysis (Figure 4C). However, the higher concentration of atorvastatin (50 µg/mL, lane 4) and both concentrations of rosuvastatin (lanes 5 and 6) significantly attenuated p-ERK levels, with rosuvastatin at 20 µg/mL again producing the most substantial reduction (*p* < 0.01). When expressed as a ratio of phosphorylated to total ERK (Figure 4D), LPS stimulation substantially increased the p-ERK:ERK ratio from approximately 1.0 to 1.7, indicating enhanced activation of the ERK signalling cascade. Both statins reduced this ratio in a concentration-dependent manner, with atorvastatin at 50 µg/mL and rosuvastatin at both concentrations restoring the ratio to near-baseline levels, suggesting effective suppression of ERK activation rather than merely reducing protein abundance.

**Figure 5:**
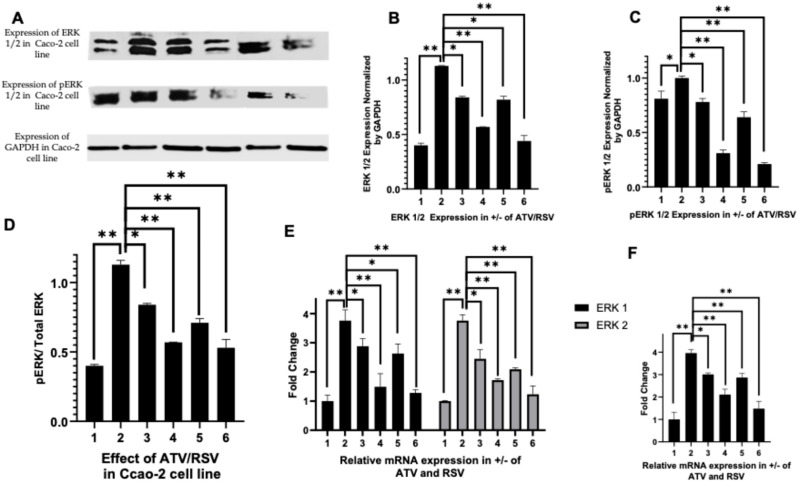
Atorvastatin and rosuvastatin suppress ERK1/2 signalling in LPS-stimulated Caco-2 colorectal cancer cells. (**A**) Representative Western blot analysis of total ERK1/2, phosphorylated ERK1/2 (pERK1/2), and GAPDH (loading control) in Caco-2 cells. Lanes represent: (1) untreated control; (2) LPS alone; (3) LPS + atorvastatin 20 µg/mL; (4) LPS + atorvastatin 50 µg/mL; (5) LPS + rosuvastatin 10 µg/mL; (6) LPS + rosuvastatin 20 µg/mL; (**B**) Densitometric quantification of total ERK1/2 expression normalised to GAPDH; (**C**) Densitometric quantification of pERK1/2 expression normalised to GAPDH. (**D**) Ratio of phosphorylated ERK to total ERK protein levels; (**E**) Quantitative RT-PCR analysis of ERK1 (MAPK3, black bars) and ERK2 (MAPK1, grey bars) mRNA expression relative to untreated controls; (**F**) Quantitative RT-PCR analysis of DUSP6 mRNA expression, a transcriptional target and negative feedback regulator of ERK signalling. (Data are presented as the mean ± s.e.m. from three independent experiments. Statistical significance was determined by one-way ANOVA followed by Tukey’s post hoc test. * *p* < 0.05, ** *p* < 0.01.)

In Caco-2 cells, the effects of statin treatment on ERK signalling were considerably more pronounced. Western blot analysis (Figure 5A) revealed that untreated Caco-2 cells exhibited very low basal expression of both total ERK1/2 and p-ERK (lane 1), whereas LPS stimulation (lane 2) induced a dramatic upregulation of both proteins. This induction was substantially greater in magnitude than that observed in HT 29 cells. Co-treatment with atorvastatin at 20 µg/mL (lane 3) and 50 µg/mL (lane 4) markedly attenuated ERK1/2 expression, reducing protein levels to approximately half of those observed following LPS stimulation. Rosuvastatin treatment produced even more substantial suppression, with the 20 µg/mL concentration (lane 6) reducing ERK1/2 expression to levels approaching those of unstimulated controls. Densitometric analysis (Figure 5B) confirmed statistically significant reductions across all treatment conditions (*p* < 0.05 to *p* < 0.01), with rosuvastatin demonstrating superior efficacy compared to atorvastatin at equivalent concentrations.

The pattern of p-ERK expression in Caco-2 cells (Figure 5A, middle panel; Figure 5C) mirrored that of total ERK1/2 but with notably greater sensitivity to statin treatment. LPS induced a robust increase in p-ERK levels (lane 2), which was progressively attenuated by atorvastatin treatment (lanes 3 and 4). Rosuvastatin exhibited particularly potent suppressive activity, with both concentrations (lanes 5 and 6) reducing p-ERK expression to barely detectable levels. The p-ERK:ERK ratio analysis (Figure 5D) demonstrated that LPS stimulation approximately tripled the activation ratio compared to baseline. Both statins reduced this ratio in a concentration-dependent fashion, with atorvastatin at 50 µg/mL and rosuvastatin at 20 µg/mL producing the most substantial reductions. Unlike the pattern observed in HT 29 cells with statin treatment, no paradoxical increase in the p-ERK:ERK ratio was observed with statin treatment in Caco-2 cells, indicating proportional downregulation of both total and phosphorylated ERK isoforms.

Transcriptional analysis by RT-PCR revealed consistent effects at the mRNA level. In HT 29 cells (Figure 4E), LPS stimulation induced approximately 3.5 to 4-fold increases in both ERK1 (MAPK3) and ERK2 (MAPK1) transcripts compared to untreated controls. Both statins suppressed ERK1 and ERK2 mRNA expression in a concentration-dependent manner, with rosuvastatin at 20 µg/mL restoring transcript levels to near-baseline values. ERK1 appeared somewhat more responsive to statin treatment than ERK2, particularly with rosuvastatin. A similar pattern was observed in Caco-2 cells (Figure 5E), where LPS-induced upregulation of ERK1 and ERK2 transcripts was progressively attenuated by increasing concentrations of both statins. Rosuvastatin again demonstrated greater potency, with the 20 µg/mL concentration producing the most substantial suppression of both isoforms.

To assess downstream consequences of ERK pathway modulation, we examined the expression of DUSP6, a dual-specificity phosphatase that functions as both a target and negative regulator of MAPK signalling. In HT 29 cells (Figure 4F), LPS stimulation induced a nearly 4-fold increase in DUSP6 mRNA expression, consistent with activation of the ERK signalling cascade. Both atorvastatin and rosuvastatin significantly reduced DUSP6 transcript levels in a concentration-dependent manner (*p* < 0.05 to *p* < 0.01), with the highest concentration of each statin producing reductions of approximately 50% relative to LPS-stimulated cells. In Caco-2 cells (Figure 5F), a similar pattern emerged, with LPS inducing robust DUSP6 upregulation that was subsequently attenuated by statin co-treatment. Rosuvastatin at 20 µg/mL produced the most pronounced suppression, reducing DUSP6 mRNA to levels only marginally above baseline.

Collectively, these findings demonstrate that both atorvastatin and rosuvastatin effectively suppress ERK1/2 signalling in LPS-stimulated HT 29 and Caco-2 colorectal cancer cells. The suppressive effects were observed at multiple levels, encompassing total protein expression, phosphorylation status, and transcriptional regulation of pathway components. Rosuvastatin consistently exhibited greater potency than atorvastatin across both cell lines, potentially reflecting differences in lipophilicity, cellular uptake, or target engagement. The coordinate suppression of total ERK, phosphorylated ERK, and the downstream target DUSP6 indicates that statins act at or upstream of ERK transcription rather than solely through post-translational mechanisms. Given the established role of ERK signalling in promoting proliferation, survival, and inflammatory responses downstream of PAR-2 in CRC, these results suggest that the previously observed suppression of PAR-2 expression by statins translates into functional attenuation of a key effector pathway, thereby reinforcing their potential therapeutic utility in inflammatory and neoplastic conditions of the colon.

### 2.5. Atorvastatin and Rosuvastatin Attenuate Tnf-A Expression in Lps-Stimulated Ht 29 and Caco-2 Cells

Having established that atorvastatin and rosuvastatin suppress ERK1/2 activation in colorectal cancer cells, we next examined TNF-α as a functionally relevant downstream effector. TNF-α represents a critical node that integrates mitogenic and inflammatory signalling outputs, being transcriptionally regulated by both MAPK and NF-κB pathways that lie downstream of PAR-2 and ERK activation. Moreover, TNF-α serves as a proximal trigger for extrinsic apoptotic signalling through caspase-8 activation and can engage mitochondrial death pathways under certain contexts. Examination of TNF-α expression therefore provides a mechanistic bridge between the upstream receptor-kinase signalling events documented above and the functional cellular responses that determine tumour cell fate.

**Figure 6:**
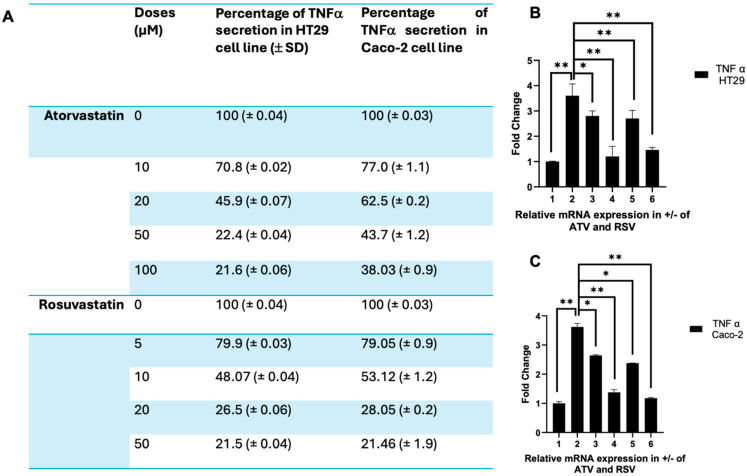
Atorvastatin and rosuvastatin suppress TNF-α expression in LPS-stimulated HT 29 and Caco-2 colorectal cancer cells. (**A**) Quantification of TNF-α protein secretion by ELISA in HT 29 and Caco-2 cells treated with increasing concentrations of atorvastatin (0, 10, 20, 50, and 100 µM) or rosuvastatin (0, 5, 10, 20, and 50 µM). Values represent percentage of TNF-α secretion relative to untreated controls (set at 100%) and are expressed as the mean ± s.d. from three independent experiments; (**B**) Quantitative RT-PCR analysis of TNF-α mRNA expression in HT 29 cells. Lanes represent: (1) untreated control; (2) LPS alone; (3) LPS + atorvastatin 20 µg/mL; (4) LPS + atorvastatin 50 µg/mL; (5) LPS + rosuvastatin 10 µg/mL; (6) LPS + rosuvastatin 20 µg/mL; (**C**) Quantitative RT-PCR analysis of TNF-α mRNA expression in Caco-2 cells under identical treatment conditions. Data in (**B**,**C**) are presented as the mean ± s.e.m. from three independent experiments. Statistical significance was determined by one-way ANOVA followed by Tukey’s post hoc test. * *p* < 0.05, ** *p* < 0.01.

To assess the effects of statins on TNF-α secretion, we performed ELISA measurements across a range of concentrations for both atorvastatin (10, 20, 50, and 100 µM) and rosuvastatin (5, 10, 20, and 50 µM) in HT 29 and Caco-2 cells (Figure 6A). In HT 29 cells, atorvastatin produced a concentration-dependent reduction in TNF-α secretion, decreasing from baseline levels to 70.8% at 10 µM, 45.9% at 20 µM, 22.4% at 50 µM, and 21.6% at 100 µM. Rosuvastatin demonstrated comparable efficacy at lower concentrations, reducing TNF-α secretion to 79.9% at 5 µM, 48.07% at 10 µM, 26.5% at 20 µM, and 21.5% at 50 µM. In Caco-2 cells, a similar pattern emerged, although the magnitude of suppression was somewhat attenuated compared to HT 29 cells. Atorvastatin reduced TNF-α secretion to 77.0% at 10 µM, 62.5% at 20 µM, 43.7% at 50 µM, and 38.03% at 100 µM. Rosuvastatin again exhibited greater potency, suppressing secretion to 79.05% at 5 µM, 53.12% at 10 µM, 28.05% at 20 µM, and 21.46% at 50 µM. Notably, rosuvastatin achieved approximately equivalent suppression to atorvastatin at roughly half the concentration across both cell lines, consistent with the enhanced efficacy observed in our ERK signalling experiments.

To determine whether these effects were reflected at the transcriptional level, we performed quantitative RT-PCR analysis of TNF-α mRNA expression in both cell lines treated with the concentrations used throughout this study (Figure 6B,C). In HT 29 cells (Figure 6B), LPS stimulation induced an approximately 3.5-fold increase in TNF-α transcripts compared to untreated controls. Co-treatment with atorvastatin at 20 µg/mL and 50 µg/mL progressively attenuated this induction, reducing TNF-α mRNA to approximately 2.8-fold and 1.1-fold of baseline, respectively (*p* < 0.05 and *p* < 0.01). Rosuvastatin treatment produced even more pronounced suppression, with 10 µg/mL reducing TNF-α transcripts to approximately 1.2-fold and 20 µg/mL achieving near-complete normalization to baseline levels (*p* < 0.01). In Caco-2 cells (Figure 6C), LPS induced a comparable increase in TNF-α mRNA of approximately 3.6-fold. Both statins suppressed this induction in a concentration-dependent fashion, with atorvastatin at 50 µg/mL and rosuvastatin at 20 µg/mL producing the most substantial reductions (*p* < 0.01). As observed with protein secretion, rosuvastatin demonstrated superior transcriptional suppression compared to atorvastatin at equivalent concentrations.

Collectively, these findings demonstrate that both atorvastatin and rosuvastatin effectively suppress TNF-α expression at both protein and mRNA levels in LPS-stimulated colorectal cancer cells. The coordinate suppression of TNF-α secretion and transcription, together with the preceding observations of attenuated ERK1/2 activation, supports a model wherein statins disrupt the PAR-2/ERK/TNF-α signalling axis. Given the established role of TNF-α in promoting inflammatory responses and modulating apoptotic sensitivity in colorectal cancer, these results suggest that the anti-inflammatory properties of statins may contribute to their reported chemopreventive effects in this malignancy.

### 2.6. Statins Induce Apoptosis in Ht29 and Caco-2 Cells via Par-2/Erk/Tnf-A-Mediated Activation of Extrinsic and Intrinsic Pathways

Having demonstrated that atorvastatin and rosuvastatin suppress PAR-2 expression, attenuate ERK1/2 phosphorylation, and downregulate TNF-α in LPS-stimulated HT29 and Caco-2 cells, we next investigated whether these upstream signalling events translate into activation of apoptotic cell death. Statins have been shown to exert pleiotropic anti-cancer effects independent of their cholesterol-lowering activity, in part through inhibition of isoprenoid intermediates required for Ras and Rho GTPase membrane localization and function [47]. Given that PAR-2 signalling promotes survival by engaging MEK1/2–ERK1/2 and PI3K–Akt pathways, which in turn suppress initiator caspase activation, upregulate anti-apoptotic Bcl-2 family members, and prevent mitochondrial outer membrane permeabilization (MOMP) [48], we hypothesized that statin-mediated suppression of the PAR-2–ERK–TNF-α axis would relieve this apoptotic blockade. To test this, we evaluated markers of both extrinsic (caspase-8, caspase-3) and intrinsic (Bcl-2, Bax) apoptotic pathways and confirmed apoptosis induction by Annexin V/PI flow cytometry.

#### 2.6.1. Statins Activate the Initiator Caspase-8 to Engage the Extrinsic Apoptotic Pathway

**Figure 7:**
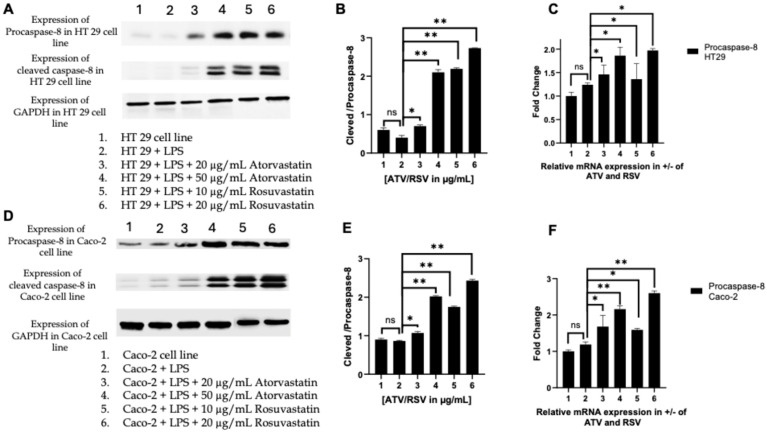
Statins activate caspase-8 and upregulate its expression in colorectal cancer cells. (**A**) Representative Western blots showing expression of pro-caspase-8 (55 kDa), cleaved caspase-8 (43/41 kDa), and GAPDH (loading control) in HT29 cells treated with LPS alone or LPS in combination with atorvastatin (20, 50 μg/mL) or rosuvastatin (10, 20 μg/mL) for 24 h; (**B**) Densitometric quantification of the cleaved caspase-8 to pro-caspase-8 ratio in HT29 cells; (**C**) RT-qPCR analysis of *CASP8* mRNA expression in HT29 cells, normalized to GAPDH and expressed as fold change relative to untreated controls. (**D**) Representative Western blots of pro-caspase-8, cleaved caspase-8, and GAPDH in Caco-2 cells under identical treatment conditions; (**E**) Densitometric quantification of the cleaved caspase-8 to pro-caspase-8 ratio in Caco-2 cells; (**F**) RT-qPCR analysis of *CASP8* mRNA expression in Caco-2 cells. Data are presented as the mean ± SEM from three independent experiments. Statistical significance was determined by one-way ANOVA followed by Dunnett’s post hoc test. * *p* < 0.05, ** *p* < 0.01 versus LPS-treated control; ns, not significant.

To determine whether statin-mediated suppression of the PAR-2–ERK–TNF-α signalling axis culminates in apoptotic commitment, we examined the activation status of caspase-8, the apical initiator caspase of the extrinsic death receptor pathway. Western blot analysis revealed that both atorvastatin and rosuvastatin induced a pronounced increase in the cleaved (active) form of caspase-8 relative to its zymogen precursor, pro-caspase-8, in LPS-stimulated HT29 cells (Figure 7A,B). Quantification of the cleaved-to-pro-caspase-8 ratio, a metric that intrinsically controls for loading variation and reflects the net activation state of the enzyme, demonstrated a dose-dependent shift toward the active form. In HT29 cells, atorvastatin at 20 and 50 μg/mL elevated this ratio approximately 1.5-fold and 2.1-fold, respectively, compared to LPS-treated controls (Figure 7B). Notably, rosuvastatin exhibited greater potency, with 10 and 20 μg/mL concentrations yielding 2.2-fold and 2.8-fold increases, respectively, achieving comparable or superior caspase-8 activation at lower molar concentrations than atorvastatin.

This pattern was recapitulated in Caco-2 cells, confirming that the observed effects are not cell line-specific (Figure 7D,E). Here, rosuvastatin at 20 μg/mL induced a 2.5-fold increase in the cleaved-to-pro-caspase-8 ratio, exceeding the 2.0-fold elevation achieved by atorvastatin at 50 μg/mL. The heightened efficacy of rosuvastatin may reflect its greater hydrophilicity and distinct pharmacokinetic profile, which could influence intracellular accumulation and target engagement in epithelial cancer cells.

Intriguingly, parallel assessment of *CASP8* transcript levels by RT-PCR revealed that statin treatment also upregulated pro-caspase-8 mRNA expression in both cell lines (Figure 7C,F). In HT29 cells, atorvastatin and rosuvastatin increased *CASP8* transcripts by 1.4- to 2.0-fold, while in Caco-2 cells, this induction ranged from 1.8- to 2.8-fold. This dual mechanism, transcriptional priming of the apoptotic machinery coupled with post-translational activation, suggests that statins sensitize CRC cells to death receptor-mediated apoptosis at multiple regulatory nodes. Importantly, LPS stimulation alone did not significantly alter caspase-8 cleavage or expression relative to untreated controls, indicating that the pro-apoptotic effects are attributable to statin intervention rather than inflammatory priming.

Collectively, these findings establish that atorvastatin and rosuvastatin engage the extrinsic apoptotic pathway through caspase-8 activation, with rosuvastatin demonstrating superior potency in both colorectal cancer models examined.

#### 2.6.2. Statins Promote Mitochondrial Priming Through Bax Induction and Bcl-2 Suppression

Activation of caspase-8 at the death-inducing signalling complex (DISC) can directly engage the executioner caspase cascade or, in Type II cells, amplify the apoptotic signal through the mitochondrial pathway via cleavage of the BH3-only protein Bid. Truncated Bid (tBid) translocates to the outer mitochondrial membrane where it promotes Bax oligomerization and MOMP, thereby integrating extrinsic and intrinsic apoptotic circuits [49]. Having established that statins activate caspase-8, we next examined whether this upstream event propagates to the mitochondrial checkpoint by assessing the expression of the pro-apoptotic effector Bax and its anti-apoptotic counterpart Bcl-2.

**Figure 8:**
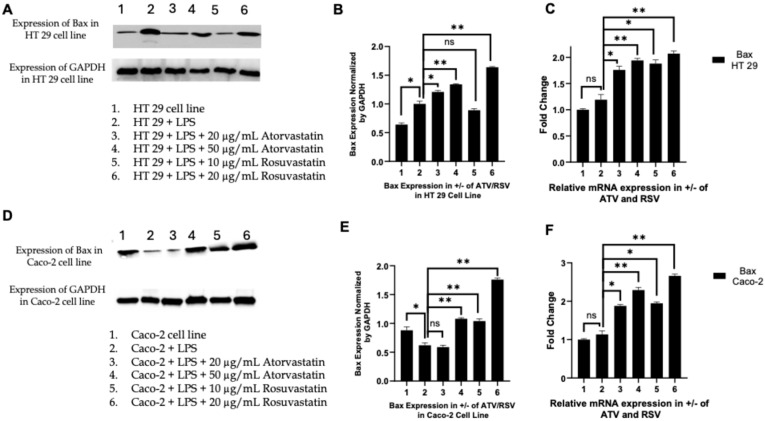
Statins induce Bax expression at protein and transcriptional levels in colorectal cancer cells. (**A**) Representative Western blots showing expression of Bax (21 kDa) and GAPDH (loading control) in HT29 cells treated with LPS alone or LPS in combination with atorvastatin (20, 50 μg/mL) or rosuvastatin (10, 20 μg/mL) for 24 h; (**B**) Densitometric quantification of Bax protein expression normalized to GAPDH in HT29 cells; (**C**) RT-qPCR analysis of *BAX* mRNA expression in HT29 cells, normalized to GAPDH and expressed as fold change relative to untreated controls; (**D**) Representative Western blots of Bax and GAPDH in Caco-2 cells under identical treatment conditions; (**E**) Densitometric quantification of Bax protein expression normalized to GAPDH in Caco-2 cells; (**F**) RT-PCR analysis of *BAX* mRNA expression in Caco-2 cells. Data are presented as the mean ± SEM from three independent experiments. Statistical significance was determined by one-way ANOVA followed by Dunnett’s post hoc test. * *p* < 0.05, ** *p* < 0.01 versus LPS-treated control; ns, not significant.

Western blot analysis revealed that LPS stimulation alone induced only marginal changes in Bax protein levels in HT29 and Caco-2 cells (Figure 8A,D). In contrast, co-treatment with atorvastatin or rosuvastatin produced a robust, dose-dependent upregulation of Bax protein expression (Figure 8B,E). In HT29 cells, atorvastatin at 20 and 50 μg/mL elevated Bax levels by approximately 1.3-fold and 1.4-fold, respectively, whereas rosuvastatin at 10 and 20 μg/mL induced more pronounced increases of 1.5-fold and 1.7-fold relative to LPS-treated controls. A similar pattern emerged in Caco-2 cells, where rosuvastatin at 20 μg/mL achieved a 1.9-fold induction of Bax, surpassing the 1.6-fold increase observed with atorvastatin at 50 μg/mL. RT-PCR analysis confirmed that this upregulation occurred at the transcriptional level, with *BAX* mRNA exhibiting 1.5- to 2.0-fold increases across both cell lines following statin treatment (Figure 8C,F). These findings indicate that statins transcriptionally prime colorectal cancer cells for mitochondrial apoptosis by enhancing Bax expression.

**Figure 9:**
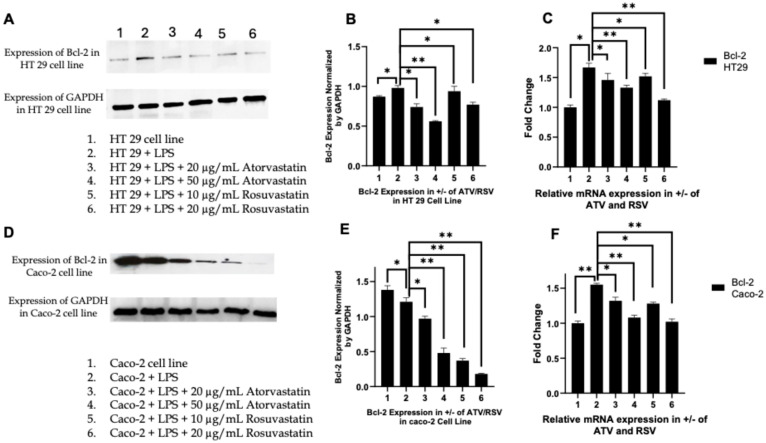
Statins suppress Bcl-2 expression at protein and transcriptional levels in colorectal cancer cells. (**A**) Representative Western blots showing expression of Bcl-2 (26 kDa) and GAPDH (loading control) in HT29 cells treated with LPS alone or LPS in combination with atorvastatin (20, 50 μg/mL) or rosuvastatin (10, 20 μg/mL) for 24 h; (**B**) Densitometric quantification of Bcl-2 protein expression normalized to GAPDH in HT29 cells; (**C**) RT-qPCR analysis of *BCL2* mRNA expression in HT29 cells, normalized to GAPDH and expressed as fold change relative to untreated controls; (**D**) Representative Western blots of Bcl-2 and GAPDH in Caco-2 cells under identical treatment conditions; (**E**) Densitometric quantification of Bcl-2 protein expression normalized to GAPDH in Caco-2 cells; (**F**) RT-qPCR analysis of *BCL2* mRNA expression in Caco-2 cells. Data are presented as the mean ± SEM from three independent experiments. Statistical significance was determined by one-way ANOVA followed by Dunnett’s post hoc test. * *p* < 0.05, ** *p* < 0.01 versus LPS-treated control.

In parallel, we assessed Bcl-2, the principal anti-apoptotic guardian of mitochondrial integrity. Consistent with a pro-survival inflammatory phenotype, LPS stimulation markedly upregulated Bcl-2 protein expression in both HT29 and Caco-2 cells (Figure 9A,D). Statin co-treatment reversed this effect in a dose-dependent manner. In HT29 cells, atorvastatin reduced Bcl-2 protein levels to approximately 0.6-fold and 0.5-fold of LPS-treated controls at 20 and 50 μg/mL, respectively, while rosuvastatin achieved reductions to 0.4-fold and 0.3-fold at 10 and 20 μg/mL (Figure 9B). The suppression was even more striking in Caco-2 cells, where rosuvastatin at 20 μg/mL diminished Bcl-2 expression to approximately 0.2-fold of LPS controls (Figure 9E). RT-PCR analysis demonstrated concordant decreases in *BCL2* transcript levels (Figure 9C,F), confirming that statins suppress the anti-apoptotic program at the transcriptional level.

Collectively, these reciprocal changes, Bax induction coupled with Bcl-2 suppression, shift the Bax/Bcl-2 ratio decisively in favour of apoptosis. This coordinated modulation of the mitochondrial checkpoint, downstream of caspase-8 activation, indicates that statins engage both extrinsic and intrinsic apoptotic pathways in a convergent pro-death program. Notably, rosuvastatin consistently demonstrated superior efficacy in modulating the Bax/Bcl-2 axis across both colorectal cancer cell lines, achieving greater pro-apoptotic shifts at lower concentrations than atorvastatin.

#### 2.6.3. Statins Activate the Executioner Caspase-3 and Transcriptionally Prime the Apoptotic Machinery

The convergence of extrinsic and intrinsic apoptotic pathways culminates in the proteolytic activation of caspase-3, the principal executioner caspase responsible for the systematic dismantling of cellular architecture. Caspase-3 exists as an inactive zymogen (pro-caspase-3, 32 kDa) that undergoes cleavage by upstream initiator caspases, caspase-8 from the extrinsic arm or caspase-9 following apoptosome assembly, to yield catalytically active p17 and p12 subunits [42,43]. Having demonstrated that statins activate caspase-8 and shift the Bax/Bcl-2 equilibrium toward mitochondrial permeabilization, we next interrogated the activation status of caspase-3 to confirm execution of the apoptotic program.

**Figure 10:**
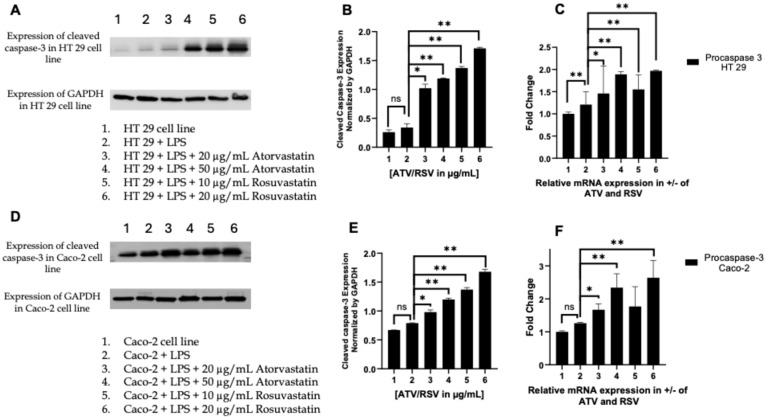
Statins induce caspase-3 cleavage and upregulate *CASP3* transcription in colorectal cancer cells. (**A**) Representative Western blots showing expression of cleaved caspase-3 (p17, 17 kDa) and GAPDH (loading control) in HT29 cells treated with LPS alone or LPS in combination with atorvastatin (20, 50 μg/mL) or rosuvastatin (10, 20 μg/mL) for 24 h; (**B**) Densitometric quantification of cleaved caspase-3 expression normalized to GAPDH in HT29 cells; (**C**) RT-qPCR analysis of *CASP3* mRNA expression in HT29 cells, normalized to GAPDH and expressed as fold change relative to untreated controls; (**D**) Representative Western blots of cleaved caspase-3 and GAPDH in Caco-2 cells under identical treatment conditions; (**E**) Densitometric quantification of cleaved caspase-3 expression normalized to GAPDH in Caco-2 cells; (**F**) RT-qPCR analysis of *CASP3* mRNA expression in Caco-2 cells. Data are presented as the mean ± SEM from three independent experiments. Statistical significance was determined by one-way ANOVA followed by Dunnett’s post hoc test. * *p* < 0.05, ** *p* < 0.01 versus LPS-treated control; ns, not significant.

Western blot analysis using a cleavage-specific antibody revealed that cleaved caspase-3 (p17) was minimally detectable in untreated HT29 and Caco-2 cells, consistent with the baseline quiescent state of the apoptotic machinery (Figure 10A,D). LPS stimulation alone failed to elicit significant caspase-3 cleavage, indicating that inflammatory signalling per se does not commit these cells to apoptotic death. In marked contrast, co-treatment with atorvastatin or rosuvastatin induced a pronounced, dose-dependent accumulation of the cleaved p17 fragment in both cell lines (Figure 10B,E). In HT29 cells, atorvastatin at 20 and 50 μg/mL elevated cleaved caspase-3 levels by approximately 1.0-fold and 1.2-fold relative to LPS-treated controls, whereas rosuvastatin achieved substantially greater activation, with 10 and 20 μg/mL concentrations producing 1.4-fold and 1.8-fold increases, respectively. This differential potency was recapitulated in Caco-2 cells, where rosuvastatin at 20 μg/mL induced a 1.9-fold increase in cleaved caspase-3—exceeding the 1.4-fold elevation observed with atorvastatin at 50 μg/mL.

Intriguingly, RT-PCR analysis revealed that statin treatment also upregulated *CASP3* transcript abundance in a dose-dependent manner (Figure 10C,F). In HT29 cells, atorvastatin and rosuvastatin induced 1.5- to 2.2-fold increases in *CASP3* mRNA, while in Caco-2 cells this transcriptional induction ranged from 1.6- to 2.8-fold at the highest concentrations tested. This finding parallels our earlier observation of *CASP8* transcriptional upregulation and suggests that statins orchestrate a coordinated transcriptional program that amplifies the apoptotic arsenal. Such dual-level regulation enhanced zymogen synthesis coupled with accelerated proteolytic activation may serve to sustain and amplify the apoptotic signal, ensuring irreversible commitment to cell death once the threshold is breached.

The robust activation of caspase-3 observed here, downstream of caspase-8 engagement and Bax/Bcl-2 modulation, provides definitive evidence that statins induce bona fide apoptotic cell death rather than alternative forms of cell demise. Moreover, the consistent superiority of rosuvastatin over atorvastatin in driving caspase-3 activation across both colorectal cancer cell lines reinforces its potential as the more efficacious agent for therapeutic exploitation in inflammation-associated colorectal malignancy.

### 2.7. Annexin V/Pi Flow Cytometry Confirms Statin Induced Apoptosis

To provide definitive confirmation of apoptotic cell death and complement the biochemical evidence obtained from caspase activation studies, Annexin V/propidium iodide (PI) dual staining flow cytometry was performed. This technique discriminates between viable cells (Annexin V−/PI−), early apoptotic cells exhibiting phosphatidylserine externalization (Annexin V+/PI−), late apoptotic cells with compromised membrane integrity (Annexin V+/PI+), and necrotic cells (Annexin V−/PI+).

**Figure 11:**
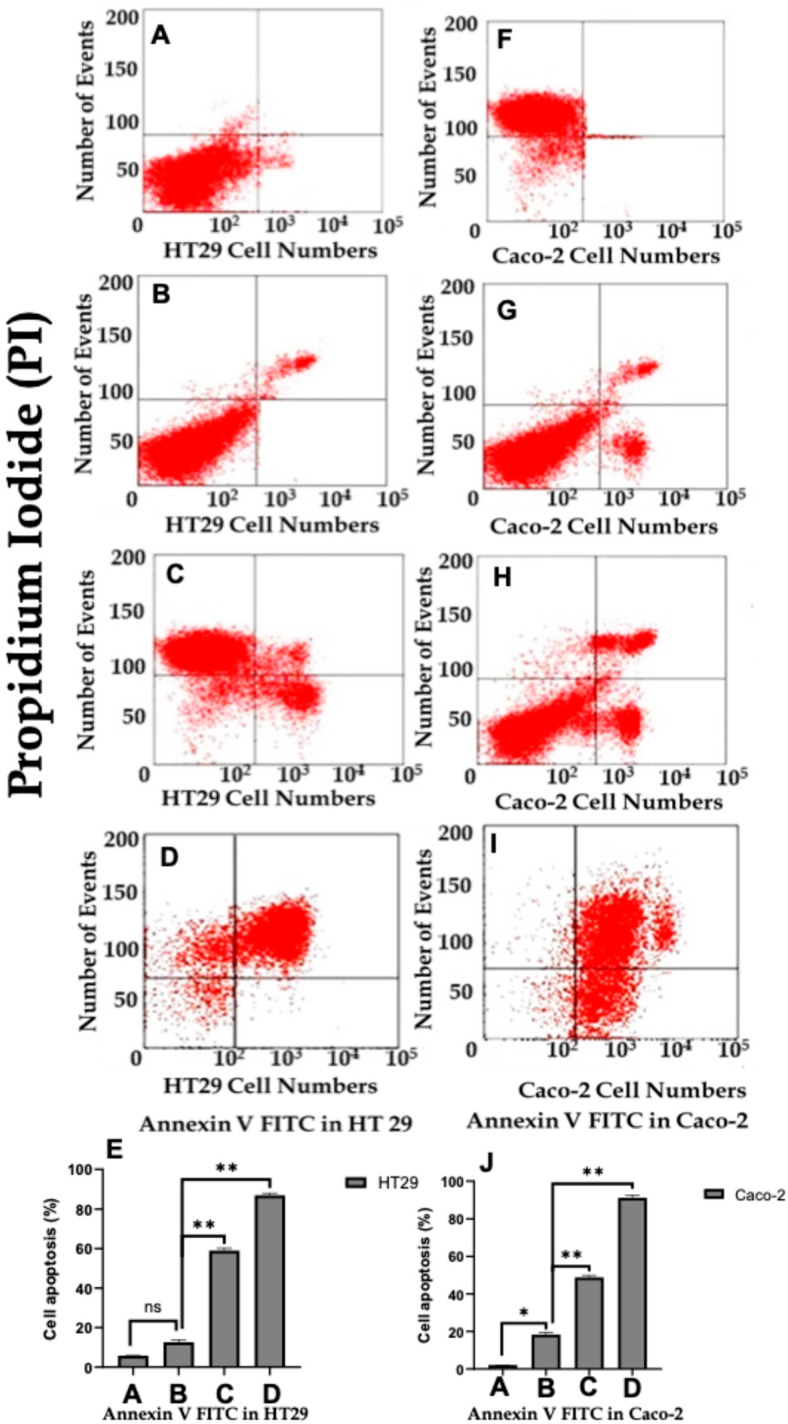
Annexin V/PI Flow Cytometry Analysis of Statin-Induced Apoptosis in Colorectal Cancer Cells. Annexin V/propidium iodide (PI) dual staining flow cytometry confirms apoptotic cell death following statin treatment in LPS-stimulated colorectal cancer cells. Representative dot plots display the distribution of cell populations across four quadrants: lower left (Annexin V−/PI−, viable cells), lower right (Annexin V+/PI−, early apoptotic cells), upper right (Annexin V+/PI+, late apoptotic cells), and upper left (Annexin V−/PI+, necrotic cells). (**A**–**E**) HT-29 cells: (**A**) Untreated control; (**B**) LPS (10 µg/mL) stimulation alone; (**C**) LPS + atorvastatin (50 µg/mL); (**D**) LPS + rosuvastatin (20 µg/mL); (**E**) Quantitative analysis of total apoptotic cells (early + late apoptosis) expressed as percentage of total cell population. (**F**–**J**) Caco-2 cells: (**F**) Untreated control; (**G**) LPS (10 µg/mL) stimulation alone; (**H**) LPS + atorvastatin (50 µg/mL); (**I**) LPS + rosuvastatin (20 µg/mL); (**J**) Quantitative analysis of total apoptotic cells expressed as percentage of total cell population. Cells were treated for 24 h prior to harvesting and staining. Data in bar graphs represent the mean ± SD from three independent experiments. Statistical significance was determined by one-way ANOVA followed by Tukey’s post hoc test. ns = Non-significant, * *p* < 0.05, ** *p* < 0.01 versus LPS control.

In HT29 cells, untreated controls displayed a predominantly viable population localized to the lower left quadrant, with minimal spontaneous apoptosis (Figure 11A). LPS stimulation alone did not substantially alter the distribution of cell populations (Figure 11B), consistent with its role as a pro-survival inflammatory stimulus. Treatment with atorvastatin (50 µg/mL) in the presence of LPS induced a discernible shift toward the early and late apoptotic quadrants (Figure 11C), indicating initiation of phosphatidylserine externalization. Notably, rosuvastatin (20 µg/mL) treatment produced a more pronounced redistribution of the cell population, with a substantial proportion of cells migrating to both the early apoptotic (lower right) and late apoptotic (upper right) quadrants (Figure 11D). Quantitative analysis revealed that rosuvastatin induced a significantly higher percentage of total apoptotic cells compared to atorvastatin (*p* < 0.01; Figure 11E).

Parallel experiments in Caco-2 cells demonstrated a similar apoptotic response pattern. Control cells exhibited high viability with negligible apoptosis (Figure 11F), and LPS exposure maintained this viable phenotype (Figure 11G). Atorvastatin treatment resulted in moderate increases in early and late apoptotic populations (Figure 11H), while rosuvastatin elicited a markedly greater apoptotic response, with conspicuous accumulation of cells in the Annexin V positive quadrants (Figure 11I). Quantification of total apoptotic cells (early plus late apoptosis) confirmed that rosuvastatin treatment significantly exceeded the apoptotic induction achieved by atorvastatin in Caco-2 cells (*p* < 0.01; Figure 11J).

These flow cytometric findings provide functional validation that the molecular alterations observed in caspase-8, Bax/Bcl-2 ratio, and caspase-3 activation culminate in bona fide apoptotic cell death. The superior capacity of rosuvastatin to drive apoptotic progression in both HT29 and Caco-2 cells corroborates its enhanced efficacy across all upstream signalling endpoints examined in this study. Collectively, these data establish that pharmacological modulation of the PAR-2/ERK/TNF-α axis by statins redirects inflammatory colorectal cancer cells from survival toward programmed cell death, with rosuvastatin demonstrating consistently greater potency than atorvastatin.

## 3. Discussion

Although statins are widely appreciated to influence oncogenic and inflammatory kinase networks, the mechanistic circuitry that links receptor proximal inflammatory sensing to tumour cell fate decisions in colorectal cancer remains incompletely resolved. In this study, we extend our prior observation that atorvastatin and rosuvastatin selectively suppress protease-activated receptor 2 expression in LPS primed colorectal cancer cells while leaving protease-activated receptor 1 unchanged, and we map the downstream signalling and fate consequences of that receptor level modulation. Specifically, in two complementary colorectal cancer epithelial models, HT 29 and Caco 2, statin exposure is associated with coordinated attenuation of ERK1 and ERK2 pathway output, including reduced ERK abundance and phosphorylation, suppression of an ERK-responsive transcriptional target, and concordant reduction in tumour necrosis factor alpha at both transcript and secreted protein levels. Within this inflammatory context, reduction in tumour necrosis factor alpha is mechanistically expected rather than paradoxical, because LPS-driven cytokine induction is amplified by receptor coupled signalling and mitogen-activated protein kinase activity, such that dampening of protease-activated receptor 2-linked ERK output is predicted to lower cytokine production. Importantly, the present work further connects this signalling trajectory to a reconstructed apoptotic programme across multiple orthogonal nodes and phenotypic validation, thereby providing an integrated framework in which statin exposure in an inflammatory colorectal cancer setting is coupled to re engagement of programmed cell death rather than persistence of inflammatory survival signalling.

The pleiotropic effects of statins extend well beyond their canonical role in cholesterol homeostasis, with accumulating evidence implicating these agents in the modulation of inflammatory signalling and tumour cell fate. In a preceding investigation, we demonstrated that atorvastatin and rosuvastatin selectively suppress PAR-2 expression in LPS-stimulated HT-29 and Caco-2 colorectal cancer cells while leaving PAR-1 expression unaffected [44], which we have also validated in this investigation (Figure 2 and Figure 3). This observation was mechanistically significant because PAR-2 functions as a critical node linking extracellular inflammatory cues to intracellular oncogenic signalling through its capacity to activate ERK1/2 and downstream pro-survival programmes [50]. The selectivity of statin action toward PAR-2, a receptor overexpressed in colorectal malignancies and associated with tumour progression, drug resistance, and poor prognosis, prompted us to interrogate the downstream functional consequences of this inhibition. The present study was therefore designed to map the signalling trajectory from PAR-2 suppression through ERK attenuation and TNF-α reduction to the ultimate activation of apoptotic cell death, thereby establishing a mechanistic framework for statin mediated conversion of inflammatory survival signals into programmed cell demise.

Our demonstration that atorvastatin and rosuvastatin suppress ERK1/2 phosphorylation in LPS-stimulated HT-29 and Caco-2 cells (Figure 4 and Figure 5) is consistent with the established role of PAR-2 as an upstream activator of the MAPK cascade. Darmoul and colleagues previously showed that PAR-2 activation by trypsin in HT-29 cells triggers matrix metalloproteinase dependent TGF-α release, leading to epidermal growth factor receptor transactivation and subsequent ERK1/2 phosphorylation [6]. Kawabata et al. extended these findings by demonstrating that PAR-2, but not PAR-1, stimulation induces MEK1/2 and ERK1/2 phosphorylation in DLD-1 CRC cells, with the MEK inhibitor PD98059 completely abolishing PAR-2 mediated proliferation [39]. Our data showing coordinate suppression of total ERK1/2, phosphorylated ERK1/2, and the downstream transcriptional target DUSP6 (Figure 4E,F and Figure 5E,F) indicate that statins act at or upstream of ERK transcription rather than solely through post-translational mechanisms. This interpretation aligns with findings from Jang et al., who demonstrated that simvastatin suppresses IGF-1R expression and inhibits phosphorylated ERK1/2 in HT-29 cells [51]. The more pronounced ERK suppression observed in Caco-2 cells relative to HT-29 cells may reflect differences in basal ERK expression and pathway dependence between these phenotypically distinct colorectal cancer models.

The attenuation of TNF-α secretion and transcription following statin treatment (Figure 6) represents a functionally significant downstream consequence of ERK pathway suppression. TNF-α occupies a central position in the inflammatory tumour microenvironment, where chronic signalling through TNFR1 typically activates NF-κB mediated pro-survival programmes rather than apoptotic cascades [52,53]. In our model, LPS induced a robust increase in TNF-α expression that was progressively reversed by both statins in a concentration dependent manner, with rosuvastatin achieving near complete normalization at 20 μg/mL. This finding parallels observations in other cancer systems; Dang et al. reported that atorvastatin suppresses inflammatory cytokines including IL-1β, IL-6, and TNF-α while downregulating EGFR/RhoA signalling in various cancer models [54]. The coordinate suppression of TNF-α at both protein and mRNA levels, together with the preceding observations of attenuated ERK1/2 activation, supports a model wherein statins disrupt the PAR-2/ERK/TNF-α signalling axis. Given the established role of TNF-α in promoting inflammatory responses and modulating apoptotic sensitivity in colorectal cancer, these results provide mechanistic insight into the reported chemopreventive effects of statins in this malignancy.

**Figure 12:**
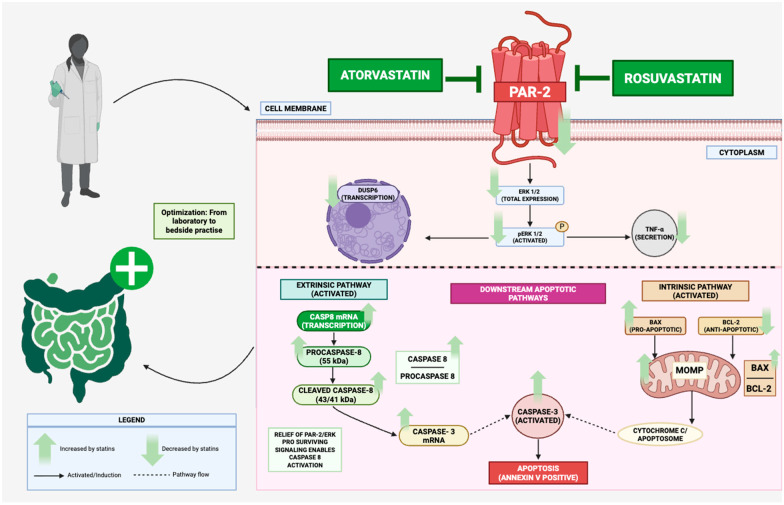
Proposed Mechanism of Statin-mediated Suppression in PAR-2/ERK signaling, Enabling Apoptosis in CRC.

Based on the collective findings of this investigation, we propose an integrated mechanistic model for statin mediated apoptosis in inflammatory colorectal cancer cells (Figure 12). At the cell membrane, both atorvastatin and rosuvastatin exert inhibitory effects on PAR-2 expression, thereby attenuating receptor level pro-survival signalling. This upstream blockade propagates to the cytoplasmic compartment, where diminished PAR-2 activity results in reduced total ERK1/2 expression and decreased phosphorylation of ERK1/2, with concurrent suppression of the transcriptional target DUSP6. The consequent attenuation of ERK signalling diminishes TNF-α secretion, thereby disrupting the autocrine and paracrine inflammatory loops that sustain colorectal cancer cell survival. Critically, relief of PAR-2/ERK mediated pro-survival signalling enables engagement of dual apoptotic cascades. The extrinsic pathway is activated through transcriptional upregulation of CASP8 mRNA, leading to increased procaspase-8 (55 kDa) synthesis and subsequent proteolytic generation of cleaved caspase-8 (43/41 kDa). Simultaneously, the intrinsic mitochondrial pathway is engaged through reciprocal modulation of Bcl-2 family proteins, wherein pro-apoptotic Bax expression is enhanced while anti-apoptotic Bcl-2 is suppressed. This altered Bax/Bcl-2 ratio promotes MOMP, facilitating cytochrome c release and apoptosome formation. Both extrinsic and intrinsic pathways converge upon caspase-3, the common executioner caspase, whose activation drives the terminal apoptotic programme. The functional culmination of this signalling cascade is validated by Annexin V positivity, confirming bona fide apoptotic cell death. This mechanistic framework positions PAR-2 as a druggable node through which statins can redirect inflammatory survival signalling toward programmed cell demise, with potential implications for translating these laboratory findings to bedside clinical practice in colorectal cancer management.

The pleiotropic biology of statins warrants explicit consideration when interpreting pathway attribution in inflammatory colorectal cancer models. Beyond mevalonate restriction, statins have been reported to modulate NF-κB-driven transcriptional programs, redox-responsive survival pathways, and broader cytokine signalling networks, any of which could theoretically influence apoptosis in parallel to PAR 2 regulation. Nevertheless, several features of our dataset argue against a purely non-specific anti-inflammatory- or stress-driven explanation. Under identical LPS priming and exposure conditions, we consistently observed selective suppression of PAR 2 at both transcript and protein levels while PAR 1 remained unchanged, supporting receptor level specificity within the protease-activated receptor family. This receptor selectivity is accompanied by a coherent downstream trajectory involving attenuation of total ERK1 and ERK2 abundance, reduced ERK phosphorylation, and suppression of the ERK-responsive transcriptional target DUSP6, together with diminished TNF α output. Importantly, apoptosis was reconstructed across orthogonal molecular nodes, encompassing caspase 8 activation, reciprocal Bax and Bcl 2 modulation, and caspase 3 engagement, with phenotypic validation by Annexin V and propidium iodide staining. Collectively, these aligned readouts support a model in which PAR 2 represents a major drug-responsive node within LPS-driven survival circuitry in colorectal cancer cells, while acknowledging that additional statin sensitive pathways may contribute in parallel.

A central finding of this investigation is that statin mediated suppression of pro-survival signalling translates into engagement of apoptotic cell death pathways. The activation of caspase-8, the apical initiator caspase of the extrinsic death receptor pathway (Figure 7), provides compelling evidence that statins relieve the apoptotic blockade imposed by PAR-2/ERK signalling. Our observation that the cleaved to pro-caspase-8 ratio increases in a dose dependent manner, reaching 2.8-fold elevation with rosuvastatin at 20 μg/mL in HT-29 cells, aligns with findings from Goc et al., who provided the first report demonstrating that simvastatin simultaneously modulates intrinsic and extrinsic pathways in prostate cancer cells [55]. In their study, simvastatin treatment resulted in increased mRNA and protein expression of TNF, Fas-L, Traf1, and cleaved caspase-8. The parallel upregulation of *CASP8* transcripts in our model (Figure 7C,F) suggests that statins sensitize CRC cells to death receptor mediated apoptosis through both transcriptional priming and post-translational activation. This dual mechanism may serve to sustain and amplify the apoptotic signal, ensuring irreversible commitment to cell death once the threshold is breached.

The reciprocal modulation of Bax and Bcl-2 observed in our study (Figure 8 and Figure 9) indicates that statins engage the intrinsic mitochondrial apoptotic pathway in parallel with extrinsic pathway activation. The dose dependent upregulation of Bax protein and mRNA, coupled with suppression of Bcl-2 expression, shifts the Bax/Bcl-2 ratio decisively in favour of mitochondrial outer membrane permeabilization and cytochrome c release. These findings are consistent with the extensive literature documenting statin effects on Bcl-2 family proteins in colorectal cancer. Spampanato et al. demonstrated that simvastatin induces overexpression of pro-apoptotic Bax and inhibition of Bcl-2 gene expression selectively in cancer cells [56]. Ahn et al. confirmed that simvastatin downregulates expression of Bcl-2, Bcl-xL, cIAP1, and cFLIP in COLO 205 and HCT 116 colorectal cancer cells [57]. The more pronounced effects of rosuvastatin on Bcl-2 suppression in Caco-2 cells, reducing expression to approximately 0.2 fold of LPS controls (Figure 9E), underscore the differential potency of these agents and suggest that rosuvastatin may be particularly effective at disabling the anti-apoptotic machinery in certain colorectal cancer subtypes.

The robust activation of caspase-3 observed downstream of caspase-8 engagement and Bax/Bcl-2 modulation (Figure 10) provides definitive evidence that statins induce bona fide apoptotic cell death rather than alternative forms of cell demise. Cheng et al. elucidated a detailed signalling cascade in HCT116 cells whereby simvastatin activates p38MAPK, leading to p53 phosphorylation, survivin suppression, and caspase activation [58]. Our findings extend this paradigm by demonstrating that the apoptotic programme initiated by statin treatment in LPS-stimulated cells involves convergence of both extrinsic and intrinsic pathways on the common executioner. The transcriptional upregulation of *CASP3* mRNA, paralleling our earlier observation of *CASP8* induction, suggests that statins orchestrate a coordinated transcriptional programme that amplifies the apoptotic arsenal. This enhanced zymogen synthesis coupled with accelerated proteolytic activation may represent a mechanism to overcome the elevated apoptotic threshold characteristic of cancer cells. The functional significance of these molecular alterations was validated by Annexin V/PI flow cytometry, which demonstrated a marked increase in early apoptotic (Annexin V+/PI−) and late apoptotic (Annexin V+/PI+) populations following statin treatment in both HT-29 and Caco-2 cells, with rosuvastatin consistently inducing a greater proportion of total apoptotic cells compared to atorvastatin (Figure 11).

The superior efficacy of rosuvastatin relative to atorvastatin across multiple endpoints in this study warrants mechanistic consideration. Although atorvastatin is comparatively lipophilic and can access cells through passive diffusion, rosuvastatin is relatively hydrophilic and typically exhibits greater dependence on transporter mediated influx. The physicochemical separation between these agents is substantial, with reported LogD values at pH 7.4 generally favouring atorvastatin over rosuvastatin, a property that would, on first principles, be expected to constrain passive cellular access of rosuvastatin in colonic epithelial models [55]. However, colorectal neoplasia is characterised by dysregulated transporter biology, and Lee et al. reported markedly increased expression of organic anion transporting polypeptide 1B3 (OATP1B3) in colon tumours relative to normal tissue, with immunohistochemical detection in the majority of colon adenocarcinomas [59]. While subsequent work suggests that colorectal cancer cells may express cancer-associated OATP1B3 variants with reduced transport activity [60], this does not exclude residual functional uptake, inter tumour heterogeneity, contribution from additional uptake systems, or culture dependent regulation of transporter expression that together can permit sufficient intracellular exposure for pharmacological activity. In parallel, net intracellular accumulation is also shaped by efflux systems. Rosuvastatin is a recognised substrate for breast cancer resistance protein (ABCG2), and transporter mediated modulation of ABCG2 can produce clinically meaningful shifts in rosuvastatin exposure, supporting the concept that differences in uptake and efflux balance may influence effective intracellular concentrations even when nominal dosing is matched [61].

Beyond membrane transport, multiple established pharmacologic determinants can plausibly account for the observed rank order of effects without implying unmeasured causality. First, intrinsic target engagement at 3 hydroxy 3 methylglutaryl coenzyme A (HMG CoA) reductase can separate downstream pathway suppression, because rosuvastatin was structure guided to achieve high inhibitory efficiency and potency per unit exposure relative to several earlier statins [6,39,50]. Second, atorvastatin efficacy in vivo is meaningfully shaped by active hydroxylated metabolites, whereas rosuvastatin undergoes limited metabolism and acts predominantly as parent drug [51,52]. In an in vitro epithelial system that does not recapitulate hepatic cytochrome rich bioactivation, drugs whose full in vivo inhibitory portfolio includes metabolite contribution can appear systematically disadvantaged, providing a parsimonious explanation for stronger apparent activity of rosuvastatin at nominally comparable concentrations. Third, differential protein binding in serum containing media can materially alter the unbound fraction available for cellular entry and target engagement. Atorvastatin is extensively protein bound, whereas rosuvastatin is less so, raising the plausibility that a higher free fraction of rosuvastatin may be available under fetal bovine serum conditions, and that the biologically relevant driver of pathway modulation is effective unbound exposure rather than nominal dose [53,54]. Finally, the downstream biology measured here, including PAR 2 attenuation, ERK suppression, and apoptosis, is mechanistically compatible with the canonical mevalonate isoprenoid depletion model in which statin mediated restriction of farnesyl and geranylgeranyl intermediates suppresses prenylation dependent signalling nodes such as Ras and Rho family GTPases that converge on ERK-driven survival circuitry, with multiple cancer model studies supporting mevalonate pathway dependence through intermediate rescue logic [55,56]. Within this framework, a statin achieving higher effective intracellular target engagement would be expected to produce deeper suppression of ERK-associated survival signalling and a more pronounced shift toward apoptotic commitment, consistent with the pattern observed for rosuvastatin in the present dataset. While intracellular uptake, transporter function, and prenylation status were not directly quantified here, these established determinants provide a mechanistically grounded context for the greater apparent potency of rosuvastatin observed across our signalling endpoints.

While the dedicated adjuvant NSABP P 5 Trial in resected stage I to II colon cancer (NCT01011478) was terminated without public results, the clinical relevance of statin pleiotropy continues to be explored. Notably, a recent trial evaluating rosuvastatin in combination with neoadjuvant chemoradiotherapy in locally advanced rectal cancer reported improved pathological responses and favourable biomarker profiles, lending translational support to the concept of statin mediated chemosensitisation in colorectal malignancies [62]. The principal clinical trials and high-quality epidemiological studies evaluating statins in colorectal neoplasia are summarised in Table 1, highlighting the persistent gap in definitive randomised adjuvant evidence.

The mechanistic findings of this study can be integrated into a coherent signalling model linking statin action to apoptotic outcome. Inhibition of HMG-CoA reductase by statins depletes mevalonate pathway intermediates, particularly geranylgeranyl pyrophosphate (GGPP), which is required for membrane localization and activation of Rho family GTPases. Alizadeh et al. provided definitive evidence that GGPP depletion, rather than cholesterol reduction, drives statin-induced apoptosis; rescue experiments demonstrated complete reversal with mevalonate and significant rescue with GGPP, but no effect of cholesterol supplementation [67]. Zhu et al. extended this mechanistic framework in HCT116 cells by showing that simvastatin increases levels of unprenylated RhoA, Rac1, and Cdc42, leading paradoxically to increased GTP loading through disrupted binding to RhoGDIα [55]. The resulting signalling cascade involves superoxide production, JNK activation, Bim-EL upregulation, and mitochondrial apoptosis. Our observation of coordinate PAR-2 suppression adds an additional regulatory layer to this model; by attenuating receptor level signalling, statins diminish the pro-survival input that normally sustains ERK activation and opposes apoptotic commitment.

The clinical implications of our findings are substantial, particularly in the context of inflammation-associated colorectal carcinogenesis. A landmark study by Sun et al. matched cohort of 10,546 inflammatory bowel disease patients and demonstrated that statin use was associated with reduced incident colorectal cancer (adjusted hazard ratio 0.76), with a striking duration dependent benefit wherein five or more years of use achieved an adjusted odds ratio of 0.38 [59]. CRC related mortality was similarly reduced (adjusted hazard ratio 0.56), with particularly pronounced effects in ulcerative colitis patients (adjusted hazard ratio 0.58). These epidemiological observations align with our mechanistic findings demonstrating that statins interrupt the inflammatory PAR-2/ERK/TNF-α axis and redirect cellular fate toward apoptosis. Li et al. conducted a meta-analysis of survival outcomes in 130,994 colorectal cancer patients and reported that pre-diagnosis statin use was associated with reduced all-cause mortality (hazard ratio 0.85) and cancer specific mortality (hazard ratio 0.82) [60]. The potential for statins to synergize with established chemotherapeutic agents is also noteworthy; Tsubaki et al. demonstrated that simvastatin enhances the antitumour effect of oxaliplatin in KRAS mutated colorectal cancer cells while potentially reducing oxaliplatin-induced peripheral neuropathy [61]. Similarly, regorafenib combined with rosuvastatin shows a strong synergistic anticancer effect in colorectal cancer, markedly inhibiting tumor cell proliferation and inducing apoptosis both in vitro and in vivo. This synergy is mediated through enhanced suppression of the MAPK/MEK-ERK signalling pathway, supporting the potential of this combination as a novel therapeutic strategy for colorectal cancer [68]. Additionally, In an AOM-induced rat model of CRC, rosuvastatin and DFMO, particularly in low-dose combination, produced marked additive chemopreventive effects, suppressing colon adenocarcinoma multiplicity by 76%, delaying tumor progression, and exhibiting no toxicity. These effects were associated with reduced tumour polyamine levels and enhanced anti-tumor immunity via increased perforin- and IFN-γ-expressing NK cells, highlighting this combination as a promising preventive strategy for high-risk individuals [69].

Our study has certain limitations that merit acknowledgment. First, the experiments were conducted exclusively in vitro using established cell lines, which may not fully recapitulate the complexity of the tumour microenvironment or patient derived tumour heterogeneity. Second, the statin concentrations employed, while consistent with prior in vitro studies, exceed typical plasma concentrations achieved with standard oral dosing, necessitating caution in extrapolating to clinical scenarios. Third, we did not directly assess mevalonate pathway intermediate depletion or protein prenylation status, which would have provided additional mechanistic insight. Furthermore, the 24-h treatment duration captures acute effects but may not reflect the consequences of chronic statin exposure relevant to chemoprevention.

It should be noted that, although the present data strongly implicate the PAR-2–ERK axis as a critical conduit linking statin treatment to apoptotic reprogramming, we did not perform genetic rescue or overexpression experiments to prove singular causality. Prior work in colonic epithelial and colorectal cancer models has established that PAR-2 activation exerts robust anti-apoptotic effects, whereas PAR-2 silencing or pharmacologic inhibition restores caspase-8/-3 activation and enhances susceptibility to cytokine- or chemotherapy-induced apoptosis [43,63,64,65]. Our observation that statin-induced downregulation of PAR-2 is consistently accompanied by ERK deactivation, suppression of TNF-α and Bcl-2, upregulation of Bax, and increased Annexin V–defined apoptosis in both HT-29 and Caco-2 cells is therefore highly congruent with this established biology. Nonetheless, given the pleiotropic nature of statins, we cannot exclude additional PAR-2–independent contributions (for example via direct interference with Ras/Rho prenylation or NF-κB signaling), and future studies incorporating PAR-2 knockdown and overexpression strategies will be essential to dissect the relative weight of receptor-dependent versus receptor-independent mechanisms.

Additionally, a limitation of the present study is that we did not quantify intracellular statin accumulation, transporter expression or functional activity, or biochemical prenylation status of small GTPases, nor did we perform mevalonate pathway intermediate rescue experiments. Accordingly, the mechanistic explanations advanced for rosuvastatin’s greater apparent potency are presented as biologically plausible, literature supported determinants rather than experimentally proven causal drivers within this dataset. Future work integrating direct intracellular exposure measurements, transporter profiling, and prenylation readouts will be important to resolve the relative contributions of disposition and isoprenoid depletion to statin mediated suppression of inflammatory survival signalling in colorectal cancer.

Moreover, a limitation of the present work is that pathway attribution is inferred from pharmacologic modulation and axis level concordance rather than formal genetic epistasis. PAR 2 knockout, PAR 2 rescue, or receptor overexpression strategies were not performed, and we did not directly quantify NF-κB activity, antioxidant response programs, or intracellular reactive oxygen species under statin exposure. Accordingly, while the selective suppression of PAR 2 with sparing of PAR 1 and the coherent downstream ERK and TNF α attenuation coupled to multi node apoptosis readouts support PAR 2 centred signalling as a major contributor, we cannot exclude additional parallel contributions arising from broader statin pleiotropy. Future work incorporating PAR 2 genetic perturbation together with pathway specific reporters will be important to refine the relative weighting of intersecting inflammatory and survival networks.

## 4. Materials and Methods

### 4.1. Study Design

This investigation was designed as a cross-sectional in vitro experimental study to characterize the acute effects of statins (atorvastatin and rosuvastatin) on the modulation of inflammatory mediators in CRC cell lines. The design enables targeted analysis of pro-inflammatory signalling pathways at a specific time point, providing mechanistic insights into statins’ bioactivity. This approach eliminates the complexities of longitudinal studies while emphasizing statins’ potential as an anti-inflammatory nutraceutical in colorectal carcinogenesis.

### 4.2. Ethics Considerations

All experiments in this study were performed exclusively in vitro using established, commercially sourced human colorectal cancer cell lines. No animal models, patient-derived tissues, identifiable biological materials, or human participants were involved. In accordance with institutional policy at Mohammed Bin Rashid University of Medicine and Health Sciences (MBRU), the study is classified as minimal risk and is exempt from full Institutional Review Board (IRB) review. Confirmation regarding exemption status may be obtained from the MBRU IRB (irb@mbru.ac.ae).

Since no human subjects or identifiable data were accessed, informed consent was not required. All procedures adhered to institutional and international ethical standards governing in vitro research using non-identifiable biological materials.

### 4.3. Cell Line Selection

HT-29 and Caco-2 human colorectal cancer cell lines were selected as complementary models to represent distinct phenotypic states of colorectal tumour biology and to provide robust platforms for interrogating inflammation-driven PAR-2 signalling. HT-29, derived from a moderately differentiated adenocarcinoma, exhibits strong proliferative capacity and heightened responsiveness to inflammatory stimuli, making it suitable for modeling advanced CRC phenotypes [70]. Caco-2, originating from a well-differentiated adenocarcinoma, undergoes spontaneous enterocyte-like differentiation and maintains stable epithelial polarity, thereby serving as an established model of early-stage CRC and epithelial barrier function [71]. Other CRC cell lines were excluded based on limitations in proliferative kinetics, consistency of differentiation, cytokine responsiveness, or insufficient reproducibility for PAR-2–focused investigations. A detailed rationale for the selection of HT-29 and Caco-2, and the exclusion of other CRC lines, has been previously published in Patnaik et al. [46], to which readers are referred for comprehensive justification.

### 4.4. Cell Culture, Maintenance, and Treatment Conditions

HT-29 (C0009004, AddexBio, San Diego, CA, USA) and Caco-2 (C0009009, AddexBio, San Diego, CA, USA) human colorectal adenocarcinoma cell lines were employed for all in vitro experiments. To ensure uniform culture conditions and optimal confluence, cells were seeded at a density of 1 × 10^7^ cells per well, a parameter consistent with established models of inflammation-associated signalling in colorectal cancer [44]. All experiments were performed in biological triplicates (*n* = 3), with each biological replicate containing multiple technical replicates to minimize intra-assay variability. Statistical analyses were conducted on aggregated datasets to ensure robustness and reproducibility of findings.

Cells were cultured under strict aseptic conditions. Cryopreserved vials were thawed rapidly at 37 °C, transferred to pre-warmed complete medium, and viability was confirmed using Trypan Blue exclusion. HT-29 cells were maintained in RPMI-1640 medium (AL028A, Himedia, Pune, India) supplemented with 10% fetal bovine serum (FBS; 16000044, Gibco, Thermo Fisher Scientific, Waltham, MA, USA), 1% penicillin–streptomycin (15140122, Gibco, Thermo Fisher Scientific, Waltham, MA, USA), and 1% L-glutamine. Caco-2 cells were cultured in high-glucose Dulbecco’s Modified Eagle Medium (DMEM; 11965092, Gibco, Thermo Fisher Scientific, Waltham, MA, USA) supplemented with 10% FBS, 1% penicillin–streptomycin, and 1% non-essential amino acids. After gentle centrifugation at 200× *g* for 5 min, cells were seeded at 1 × 10^7^ cells/mL into T-75 flasks and incubated at 37 °C with 5% CO_2_. Cultures were examined daily for morphology and confluence, with the medium replaced every 48–72 h. To maintain cells in logarithmic growth, subculturing was performed at 70–80% confluence.

For subculturing, cells were washed with phosphate-buffered saline (PBS; pH 7.4) (10010023, Gibco, Thermo Fisher Scientific, Waltham, MA, USA) and detached using 0.25% Trypsin–EDTA (15400054, Gibco, Thermo Fisher Scientific, Waltham, MA, USA) for 2–4 min at 37 °C, with microscopic monitoring to avoid over-digestion. Trypsinization was neutralized with the appropriate complete medium, and cells were collected by centrifugation at 200× *g* for 5 min. Pelleted cells were resuspended and reseeded at a 1:3 split ratio in fresh T-75 flasks and incubated at 37 °C with 5% CO_2_.

For long-term preservation, both cell lines were cryopreserved following standard protocols. Cells at 70–80% confluence were harvested, resuspended in freezing medium consisting of complete culture medium supplemented with 5% dimethyl sulfoxide (DMSO), and adjusted to a density of 1 × 10^6^ cells/mL. Aliquots of 1 mL were transferred to sterile cryogenic vials (5000-0020, Gibco, Thermo Fisher Scientific, Waltham, MA, USA) and subjected to controlled-rate freezing at −1 °C/min using Mr. Frosty™ (Thermo Fisher Scientific, Waltham, MA, USA.) before being transferred to the liquid nitrogen storage system at −196 °C. This protocol ensured high post-thaw viability, genomic stability, and preservation of key phenotypic characteristics relevant to downstream assays involving lipopolysaccharide (LPS, Thermo Fisher Scientific, Waltham, MA, USA) stimulation and subsequent statin treatment (atorvastatin and rosuvastatin).

### 4.5. Assessment of Cytotoxicity Using the MTT Assay

The cytotoxicity of lipopolysaccharide (LPS; 00-4976-03, Thermo Fisher Scientific, Waltham, MA, USA) toward HT-29 and Caco-2 colorectal cancer cells was evaluated using the 3-(4,5-dimethylthiazol-2-yl)-2,5-diphenyltetrazolium bromide (MTT) assay, following established methodologies and as previously described in Patnaik et al. [44]. Mitochondrial metabolic activity, serving as a quantitative surrogate for cell viability, was assessed using the commercially available MTT reagent (M6494, Thermo Fisher Scientific, Waltham, MA, USA). Cells were exposed for 24 h to a stepwise concentration series of LPS (1, 10, 20, and 40 µg/mL), after which formazan formation was quantified spectrophotometrically at 570 nm. We selected 10 µg/mL LPS to induce a reproducible inflammatory phenotype in colorectal epithelial models while avoiding nonspecific cytotoxicity. This concentration is widely used in Caco 2 and HT 29 based epithelial inflammation paradigms and has been reported to elicit robust LPS-responsive signalling outputs in these systems. Consistent with this literature, our dose selection was empirically verified by MTT and morphology, demonstrating preserved metabolic viability across the tested exposure range and supporting 10 µg/mL as an inflammation inducing yet non-cytotoxic working concentration for downstream mechanistic assays [72,73,74]. Viability was calculated relative to untreated controls, allowing for the identification of LPS concentrations that maintained non-cytotoxic profiles suitable for subsequent inflammation-induction experiments.

### 4.6. Rationale for Selection of Atorvastatin and Rosuvastatin

The selection of atorvastatin and rosuvastatin for this investigation was driven by their complementary pharmacological properties, global clinical relevance, and mechanistic suitability for dissecting inflammation-driven signaling in CRC. These two agents represent the most widely prescribed statins worldwide [75], ensuring that any mechanistic insights derived from this study bear immediate translational resonance for patient populations frequently affected by both hyperlipidemia and heightened CRC risk. Importantly, atorvastatin and rosuvastatin occupy opposite ends of the physicochemical spectrum, atorvastatin being moderately lipophilic and readily permeable across biological membranes, whereas rosuvastatin is highly hydrophilic and preferentially taken up through organic anion transporting polypeptides (OATP1B1/1B3) [76]. This duality enables investigation of whether modulation of PAR-2–ERK–TNF-α inflammatory circuitry is contingent upon membrane diffusion, transporter-mediated uptake, or reflects a class-wide consequence of HMG-CoA reductase inhibition within colonic epithelial cells.

From a pharmacokinetic standpoint, atorvastatin exhibits a logP of approximately 4.0, high protein binding (>98%), and a prolonged elimination half-life of ~14 h [77]. Although its absolute bioavailability is modest (~14%), atorvastatin achieves substantial luminal concentrations in the gastrointestinal tract following oral dosing, thereby allowing direct contact with intestinal epithelial cells before systemic absorption. Its lipophilicity facilitates robust intracellular accumulation within colonocytes, enabling effective depletion of isoprenoid intermediates required for RAS and RHO prenylation. This makes atorvastatin particularly suited to probing prenylation-dependent inflammatory pathways such as PAR-2–ERK activation. Rosuvastatin, conversely, is characterized by a logP of ~1.4, a bioavailability approaching 75%, and a long half-life of ~19 h [78]. Although hydrophilic, rosuvastatin’s strong affinity for OATP transporters and luminal stability confer excellent accessibility to colonic epithelial cells. A significant proportion of orally administered rosuvastatin remains unmodified prior to hepatic uptake, ensuring predictable tissue exposure and minimal metabolic variability. Collectively, these features allow both statins to reach biologically relevant intracellular concentrations in the colon, thereby justifying their selection as exemplary pharmacologic probes for inflammation-linked CRC signaling.

Several clinically used statins were excluded due to suboptimal characteristics for mechanistic dissection in colonic epithelium. Simvastatin and lovastatin, although highly lipophilic, exist as lactone prodrugs that require metabolic activation and exhibit exceptionally low bioavailability (<5%), accompanied by high interindividual variability in systemic exposure, which reduces reproducibility in in vitro systems. Fluvastatin, though moderately lipophilic, undergoes rapid and extensive CYP2C9-mediated metabolism with a short half-life of 1–3 h, limiting sustained intracellular exposure. Pravastatin, the most hydrophilic statin, exhibits extremely limited membrane permeability and only ~20% absorption, rendering epithelial uptake inefficient. Pitavastatin, although pharmacologically interesting, has limited global clinical use and lacks a robust body of CRC-specific mechanistic literature, thereby diminishing its translational relevance for CRC repurposing studies. In contrast, atorvastatin and rosuvastatin offer the optimal balance of high colonic exposure, well-defined pharmacokinetics, potent inhibition of the mevalonate pathway, and extensive clinical utilization, supporting their preferential selection for interrogating LPS-induced inflammatory signaling in CRC.

These considerations are quantitatively summarized in Table 2, which compares the physicochemical, pharmacokinetic, metabolic, and transporter-related properties of all major clinical statins, highlighting the unique suitability of atorvastatin and rosuvastatin for mechanistic studies on the colonic epithelium [79]. Their complementary permeability profiles, predictable intracellular handling, and established anti-inflammatory signatures make them ideal candidates for elucidating statin-driven modulation of PAR-2–ERK–TNF-α pathways in colorectal carcinogenesis.

For all in vitro treatments, rosuvastatin was solubilized in phosphate-buffered saline (PBS) to ensure optimal stability and dispersion in an aqueous medium. In contrast, atorvastatin required initial dissolution in dimethyl sulfoxide (DMSO) before being diluted in PBS. All atorvastatin working solutions were formulated such that the final DMSO concentration did not exceed 0.1% (*v*/*v*) in the assay mixture, thereby maintaining solvent uniformity and preventing DMSO-related cytotoxic effects.

### 4.7. Induction of Inflammation in Ht-29 and Caco-2 Cells Using LPS and Statin Treatment

To establish a robust in vitro model of inflammation-driven colorectal carcinogenesis, HT-29 and Caco-2 cells were stimulated with lipopolysaccharide (LPS; 10 µg/mL), using the optimized conditions previously validated by Patnaik et al. [44]. This concentration reliably activates Toll-like receptor 4 (TLR4) in epithelial systems and induces a reproducible inflammatory signature characterized by NF-κB activation and TNF-α secretion, while avoiding nonspecific cytotoxicity. LPS was selected over direct PAR-2 agonists, as it more faithfully reproduces the inflammatory milieu relevant to colorectal carcinogenesis. TLR4 engagement amplifies endogenous protease production, including tryptase-β, neutrophil elastase, and MMP-9, and enhances transcriptional upregulation of PAR-2 itself, thereby recapitulating the well-established bidirectional TLR4–PAR-2 axis in colonic epithelial inflammation. This strategic choice circumvents the methodological limitations of trypsin and PAR-2 synthetic peptides, which are prone to proteolytic promiscuity, rapid degradation, receptor desensitization, and a lack of physiological inflammatory context, as detailed in our prior work.

Following the establishment of LPS-induced inflammation, cells were treated with defined concentrations of atorvastatin and rosuvastatin to interrogate statin-mediated modulation of the inflammatory cascade. For HT-29 cells, the experimental conditions comprised the following: untreated control, LPS alone, LPS + 20 µg/mL atorvastatin, LPS + 50 µg/mL atorvastatin, LPS + 10 µg/mL rosuvastatin, and LPS + 20 µg/mL rosuvastatin. Identical treatment groups were applied to Caco-2 cells, enabling cross-line validation of statin effects on epithelial inflammatory signaling. These concentrations were selected based on the dosing framework established in Patnaik et al. [44] and reflect exposure ranges that elicit reproducible modulation of downstream inflammatory biomarkers without impairing basal cell viability.

This experimental configuration provides a controlled platform to evaluate how atorvastatin and rosuvastatin interface with LPS-driven TLR4–PAR-2 crosstalk, and to assess their potential to attenuate proinflammatory signaling and re-engage apoptotic pathways in colorectal cancer epithelial cells. The dual-line approach further strengthens mechanistic insights by confirming that observed pharmacologic effects are not cell-line–restricted but represent conserved responses across distinct CRC phenotypic backgrounds.

### 4.8. Rna Extraction, Cdna Synthesis, and Quantitative Real-Time Pcr (Qpcr)

Total RNA was isolated from untreated control, LPS-stimulated, and statin-treated HT-29 and Caco-2 cells using the Total RNA Isolation Kit (MB13402, NZYTech, Lisbon, Portugal), following the methodological framework previously established [44]. Cells subjected to LPS-induced inflammation (10 µg/mL) and subsequently treated with atorvastatin (20 or 50 µg/mL) or rosuvastatin (10 or 20 µg/mL) underwent parallel RNA extraction to ensure uniformity of experimental processing. RNA purity and integrity were rigorously assessed using a Nanodrop Spectrophotometer (Thermo Scientific, Waltham, MA, USA). Only samples with A260/A280 ratios between 1.8 and 2.0 were advanced to downstream applications, thereby ensuring high-fidelity amplification and minimizing contamination from proteins or organic residues.

High-quality RNA samples were reverse-transcribed into complementary DNA (cDNA) using the First-Strand cDNA Synthesis Kit (NP100042, OriGene, Heidelberg, Germany), in strict accordance with the manufacturer’s protocol. The resulting cDNA served as the template for quantitative real-time polymerase chain reaction (qPCR), enabling precise profiling of transcriptional changes induced by statin exposure under inflammatory conditions.

qPCR analyses were performed on a QuantStudio 5 Flex Real-Time PCR System (Applied Biosystems, Waltham, MA, USA) using SYBR Green chemistry to quantify the expression of key inflammatory, apoptotic, and signaling genes: PAR-1, PAR-2, TNF-α, BCL-2, BAX, CASP3, CASP8, ERK1, ERK2, DUSP6, and GAPDH. All primers were designed using OriGene’s proprietary primer design algorithm, incorporating optimization for melting temperature, GC content, and predicted secondary structure minimization. Primer specificity was validated through exhaustive BLAST alignment using NCBI BLAST+ (blastn, version 2.13.0), with all primer pairs demonstrating E-values of 0.0, confirming perfect sequence correspondence with the human genome. Thermodynamic modeling, including hairpin/dimer prediction and ΔG analysis, was conducted using Primer-BLAST and OligoAnalyzer, ensuring high-efficiency amplification and reproducible Ct values across biological replicates.

The sequence characteristics, accession numbers, and bioinformatic metrics of all qPCR primers are presented in Table 3, reflecting the rigorous computational and empirical validation underlying the selection process.

Quantitative PCR reactions were performed in technical triplicates, incorporating no-template controls to exclude nonspecific amplification or environmental contamination. Thermal cycling consisted of an initial denaturation at 95 °C followed by 40 cycles of denaturation, primer-specific annealing, and extension, culminating in a high-resolution melt-curve analysis to verify amplicon specificity. Relative transcript abundance was calculated using the 2^−ΔΔCt^ method, employing GAPDH as the internal normalization control. ΔCt values were normalized to GAPDH, while ΔΔCt values were referenced against untreated baseline controls, designated as 1.0. Amplification efficiency for each primer pair was confirmed through standard-curve analyses generated from serial cDNA dilutions, with all targets demonstrating 90–110% efficiency and correlation coefficients (R^2^) exceeding 0.99. Together, these parameters ensured quantitative rigor, analytic reproducibility, and high-fidelity detection of statin-induced transcriptional alterations under inflammatory conditions.

### 4.9. Western Blot Analysis of Par-2 Signaling and Apoptotic Pathways

Western blotting was performed to delineate the modulatory effects of atorvastatin and rosuvastatin on the PAR-2–centered inflammatory axis and associated apoptotic pathways in LPS-stimulated HT-29 and Caco-2 colorectal cancer cells. All procedures adhered to the optimized workflow previously established by Patnaik et al. [46], with minor modifications appropriate to the statin-based framework employed in this study. Following LPS stimulation and subsequent statin exposure, total cellular proteins were extracted using ice-cold lysis buffer (0.5% SDS, 50 mM Tris-HCl, pH 7.4) supplemented with protease (78429, Thermo Fisher Scientific, Waltham, MA, USA) and phosphatase inhibitors (78420, Thermo Fisher Scientific, Waltham, MA, USA). Lysates were clarified by centrifugation at 12,000× *g* for 15 min at 4 °C, and protein concentrations were quantified using the bicinchoninic acid (BCA) assay kit (A55865, Thermo Fisher Scientific, Waltham, MA, USA).

Equal amounts of protein (20 µg per lane) were resolved on SDS-polyacrylamide gels and transferred onto 0.45 µm nitrocellulose membranes (1620115, Bio-Rad, Mississauga, ON, Canada) using a wet-transfer system. Membranes were blocked for 1 h at 4 °C in 3% bovine serum albumin (BSA) prepared in Tris-buffered saline (TBS), followed by overnight incubation at 4 °C with the following primary antibodies: PAR-2 (6976T, Cell Signaling Technology, Danvers, MA, USA), PAR-1 (79109T, Cell Signaling), total ERK1/2 (83533-1-RR, Proteintech, Minneapolis, MN, USA), phospho-ERK1/2 (Thr202/Tyr204) (20582-1-AP, Proteintech), cleaved caspase-3 (25128-1-AP, Proteintech), pro-caspase-8 (13423-1-AP, Proteintech), cleaved caspase-8 (9496T, Cell Signaling), BCL-2 (3498T, Cell Signaling), BAX (50599-2-Ig, Proteintech), and GAPDH (2118T, Cell Signaling), the latter used as the internal loading control. All antibodies were used at manufacturer-recommended dilutions in SuperBlock-TTBS buffer (Thermo Fisher Scientific). After extensive washing, membranes were incubated with HRP-conjugated secondary antibodies (1:2000 dilution, Cell Signaling) for 1 h at room temperature and developed using the SuperSignal ULTRA Chemiluminescent Substrate (Pierce, Thermo Fisher Scientific). Signals were visualized on Kodak Biomax films (GE Healthcare, Chicago, IL, USA) to ensure high-fidelity band capture.

Densitometric analysis was performed using ImageJ (v1.54, National Institutes of Health), employing integrated density measurements with background subtraction for quantitative precision. Band intensities for each target protein were normalized to GAPDH to correct for loading variability. Normalized values were expressed as fold changes relative to untreated controls. All experiments were conducted in biological triplicates (*n* = 3), each comprising multiple technical replicates. Statistical analyses were performed using one-way analysis of variance (ANOVA), followed by Tukey’s post hoc test (GraphPad Prism v9.0, GraphPad Software, San Diego, CA, USA), with *p* < 0.05 considered statistically significant. This analytic framework ensured that both inter-experimental variability and assay reproducibility were rigorously accounted for.

Collectively, this immunoblotting strategy provided a comprehensive evaluation of how atorvastatin and rosuvastatin modulate receptor-level signaling, downstream MAPK pathway activation, and both intrinsic and extrinsic apoptotic mechanisms in colorectal cancer cells under inflammatory conditions. The assay’s high sensitivity, validated antibody panel, and stringent quantitative procedures enabled precise mechanistic mapping of statin-mediated attenuation of PAR-2 signaling and reactivation of programmed cell death pathways.

### 4.10. Quantification of TNF-α Secretion by ELISA Following Statin Treatment

To determine the impact of atorvastatin and rosuvastatin on LPS-driven pro-inflammatory cytokine production, secreted TNF-α levels were quantified in conditioned media harvested from HT-29 and Caco-2 cells following exposure to LPS and subsequent statin treatment. Post-treatment supernatants were collected and clarified by centrifugation at 3000 rpm for 10 min at 4 °C to remove cellular debris and ensure sample integrity. TNF-α concentrations were measured using a high-sensitivity sandwich ELISA kit (ab181421, Abcam, Cambridge, MA, USA), which employed pre-coated 96-well plates with immobilized monoclonal anti–TNF–α antibodies to ensure high analytical specificity.

Assays were conducted according to the manufacturer’s protocol, with absorbance measured at 450 nm and background correction performed at 620 nm using a Hidex microplate reader (Turku, Finland). All measurements were performed in triplicate to ensure analytical precision and reproducibility. This approach enabled robust quantification of statin-mediated suppression of TNF-α secretion under inflammatory stress, providing a critical biochemical readout of how atorvastatin and rosuvastatin modulate the TLR4–PAR-2–ERK axis in colorectal cancer epithelial cells.

### 4.11. Annexin V–FITC and Propidium Iodide (PI) Dual Staining for Quantitative Apoptosis Analysis

Apoptotic responses to atorvastatin and rosuvastatin were quantified using dual Annexin V–FITC/PI staining (BMS500FI-300, Thermo Fisher Scientific, Waltham, MA, USA) followed by multiparameter flow cytometric analysis. This approach enables high-resolution discrimination among viable cells, early apoptotic cells, late apoptotic/secondary necrotic cells, and primary necrotic populations based on phosphatidylserine externalization and membrane permeability. HT-29 and Caco-2 cells (3 × 10^5^ per well in 6-well plates) were cultured to approximately 70–80% confluence, primed with LPS (10 µg/mL for 24 h), and subsequently exposed to atorvastatin (20 or 50 µg/mL) or rosuvastatin (10 or 20 µg/mL) for an additional 24 h under standardized conditions. Both adherent and non-adherent fractions were collected by gentle trypsinization, pelleted by centrifugation at 300× *g* for 5 min at 4 °C, washed twice in cold phosphate-buffered saline (PBS), and resuspended in 100 µL of 1× binding buffer.

Cells were stained with 5 µL Annexin V–FITC and 5 µL PI for 15 min at room temperature in the dark, followed by dilution in 400 µL of binding buffer. Flow cytometric acquisition was performed immediately using a BD FACSCanto II cytometer (BD Biosciences, San Jose, CA, USA) equipped with a 488-nm laser. Emission signals were collected at 530 nm (FITC) and 617 nm (PI), with compensation matrices and quadrant gating established using appropriate unstained, Annexin V-only, and PI-only controls to ensure spectral purity and accurate population discrimination. A minimum of 10,000 gated events per sample were recorded to ensure statistical robustness. Data were analyzed using FlowJo v10.9.1 (BD Biosciences), and apoptotic indices were quantified as the proportions of early apoptotic (Annexin V^+^/PI^−^), late apoptotic (Annexin V^+^/PI^+^), necrotic (Annexin V^−^/PI^+^), and viable (Annexin V^−^/PI^−^) cells.

All experiments were conducted in biological triplicate (*n* = 3), and results were reported as the mean ± SEM. Statistical significance was assessed using one-way ANOVA followed by Tukey’s post hoc test in GraphPad Prism v9.0 (GraphPad Software, San Diego, CA, USA), with *p* < 0.05 considered statistically significant. This analytical framework enabled precise quantification of statin-induced apoptotic responses under inflammatory conditions and provided independent validation of the pro-apoptotic signatures observed in the molecular assays.

### 4.12. Global Statistical Analysis

All statistical analyses were performed with strict adherence to assumptions of parametric testing and in accordance with best practices for quantitative molecular biology. Data from all experimental platforms, including qPCR, Western blot densitometry, ELISA, and Annexin V–FITC/PI flow cytometry, were acquired from a minimum of three independent biological replicates (*n* = 3), each incorporating multiple technical replicates to ensure analytic robustness and mitigate intra-assay variability. Prior to hypothesis testing, the datasets were evaluated for normality using the Shapiro–Wilk test and visually inspected through Q–Q plots to confirm their suitability for parametric methods. Homogeneity of variances across treatment groups was verified using Levene’s test.

For multi-group comparisons, statistical significance was assessed using one-way analysis of variance (ANOVA), followed by Tukey’s post hoc correction to control for familywise error across multiple pairwise contrasts. For qPCR data, relative gene expression values were calculated using the 2^−ΔΔCt^ method, with log transformation applied where appropriate to satisfy linearity and variance equality assumptions. Western blot and ELISA data were analyzed using normalized, background-corrected intensity values, while flow cytometry data were evaluated based on proportional event distributions extracted from FlowJo analysis gates.

All results are reported as the mean ± standard error of the mean (SEM) unless otherwise specified. Outliers, when present, were evaluated using robust z-score criteria and retained unless they were attributable to a technical artifact. Statistical computations were performed using GraphPad Prism v9.0 (GraphPad Software, San Diego, CA, USA). A two-tailed *p*-value < 0.05 was considered statistically significant throughout. This analytic framework ensured stringent control of experimental variability, preservation of statistical power, and high-confidence interpretation of the biological effects of atorvastatin and rosuvastatin under inflammatory conditions.

## 5. Conclusions

In conclusion, our findings support a model in which atorvastatin and rosuvastatin, under LPS-driven inflammatory priming in colorectal cancer cells, are associated with selective downregulation of protease-activated receptor 2 with sparing of protease-activated receptor 1, accompanied by attenuation of ERK pathway output and reduced tumour necrosis factor alpha production, together with multi node engagement of apoptotic signalling and Annexin V confirmed cell death. While statin pleiotropy may also influence intersecting survival programmes, the receptor selectivity and axis level concordance observed here position protease-activated receptor 2-linked signalling as a major drug-responsive node in inflammation coupled colorectal cancer cell survival. These data provide mechanistic support for further translational evaluation of statins as pathway-modulating agents in inflammation-associated colorectal neoplasia, including in rational combinations designed to exploit vulnerability created by suppression of receptor coupled mitogen-activated protein kinase survival signalling.

## Figures and Tables

**Table 1:** Epidemiological studies evaluating statins in colorectal neoplasia.

Design & Population	Study/Reference	Statin (s)	Exposure/Intervention	Primary Outcome (s)	Main Findings	Limitations/Remarks
Phase III—patients with resected stage I/II colon cancer (post-surgery)	NSABP P-5 (NCT01011478)	Rosuvastatin	Adjuvant rosuvastatin vs. placebo after surgery	Disease-free survival (DFS), overall survival (OS)	Trial terminated; no public outcome data	Lack of publicly available results; unknown adherence; underpowered
Population-based cohort, 2697 incident CRC patients, Southern Germany	Hoffmeister, Michael et al. [63]	Mixed statins (real-world users)	Pre-diagnosis statin use	Overall and CRC-specific survival, recurrence-free survival	No significant association with reduced overall or CRC-specific mortality; possible better recurrence-free survival among stage I–II statin users.	Observational, potential confounding, lack of molecular subtype stratification
Prospective cohort: high-risk patients under colonoscopic surveillance	Bertagnolli, Monica M et al. [64]	Statin use vs. non-use	Regular statin usage	Incidence of colorectal adenomas and cancers	Statin use was not associated with reduced risk; suggestion of slightly increased adenoma risk in some subgroups.	Focus on high-risk population; surveillance bias; limited power for cancer endpoint
Systematic review/meta-analysis (40 studies: RCTs, cohort, case-control)	Lytras, Theodore et al. [65]	Various statins	Long-term use vs. non-use	CRC incidence	Modest (~10%) relative risk reduction in CRC among observational studies; no significant reduction in RCTs	Heterogeneity across studies; publication bias; statin type/duration variability; lack of RCT confirmation
Meta-analysis of retrospective and cohort studies (≈40,000+ CRC patients)	Li, Liusheng et al. [66]	Mixed statins	Statin use post-diagnosis vs. no use	Overall mortality and CRC-specific mortality	Statin use associated with a significant reduction in both overall mortality (HR ≈ 0.81) and CRC-specific mortality (HR ≈ 0.78)	Potential confounding; heterogeneity (I^2^ ≈ 60%); variable statin exposure/duration; observational design
Prospective phase II RCT—locally advanced rectal cancer (neoadjuvant chemoradiotherapy)	Patil, Prachi S et al. 2025, [57]	Rosuvastatin (adjunct) + CRT vs. CRT alone	Concurrent use during neoadjuvant CRT	Pathological complete response (pCR), biomarker profile, tumour regression	Addition of rosuvastatin associated with improved pathological response rate and favourable biomarker shifts (E-CRT)	Single-centre; small sample size; short follow-up; results yet to be validated in larger/multicentre RCT

**Table 2:** Physicochemical, pharmacokinetic, metabolic, and transporter-related properties of all major clinical statins, highlighting the unique suitability of atorvastatin and rosuvastatin.

Property	Atorvastatin	Rosuvastatin	Simvastatin	Lovastatin	Fluvastatin	Pravastatin	Pitavastatin
Lipophilicity (relative)	+++	+	+++++	+++++	+++	+	++
Absorption (%)	~30	~20	60–85	30	24	~20	~50
Bioavailability (%)	~14	~75	<5	<5	24–30	~17	~50–60
Protein Binding (%)	>98	~88	>95	>98	~98	43–50	~96
Half-life (h)	14	19	2–5	2–5	1–3	1.5–2	11
Metabolism (Primary CYP)	3A4	Minimal (CYP2C9 minor)	3A4	3A4	2C9	Minimal	2C9
Hepatic Extraction (%)	Moderate	Moderate	>80	>80	Moderate	Low	Moderate
Substrate for OATP1B1	+	+++	+	?	+	+	+
Substrate for BCRP	+	+++	?	?	?	+	?
Substrate for MDR1 (P-gp)	+	+	+	+	+	−	+
Colonic Epithelial Uptake	High (passive diffusion)	High (OATP-mediated)	Low–Moderate	Low–Moderate	Low	Low	Moderate
Rationale for Inclusion	High epithelial penetration; strong prenylation inhibition	High potency; stable luminal exposure; predictable PK	Variable activation; not ideal for mechanistic clarity	Low bioavailability; high variability	Short half-life; rapid clearance	Very poor uptake	Limited global use; less CRC data

Footnote: Legend: +++++ = most lipophilic; +++ = slightly lipophilic; ++ = slightly hydrophilic; + = most hydrophilic; PK = pharmacokinetics; OATP = organic anion transporting polypeptide; BCRP = breast cancer resistance protein; MDR1/P-gp = multidrug resistance protein 1.

**Table 3:** Sequence information, accession numbers, and in silico validation metrics for qPCR primers.

Gene (Protein Function)	Pathway	Primer Direction	Sequence (5′→3′)	Accession No.	E-Value	Bit Score	Amplicon Size (bp)
BAX (Pro-apoptotic)	Apoptosis	Forward Reverse	TCAGGATGCGTCCACCAAGAAG TGTGTCCACGGCGGCAATCATC	NM_004324	0.0	1412	150
BCL-2 (Anti-apoptotic)	Apoptosis	Forward Reverse	ATCGCCCTGTGGATGACTGAGT GCCAGGAGAAATCAAACAGAGGC	BC027258	0.0	2704	174
CASP-3 (Executioner caspase)	Apoptosis	Forward	GGAAGCGAATCAATGGACTCTGG	NM_004346	0.0	2645	198
		Reverse	GCATCGACATCTGTACCAGACC				
CASP-8 (Initiator caspase)	Apoptosis	Forward	AGAAGAGGGTCATCCTGGGAGA	NM_001080125	0.0	2929	162
		Reverse	TCAGGACTTCCTTCAAGGCTGC				
ERK1 (MAPK1)	MAPK/ERK	Forward	TGGCAAGCACTACCTGGATCAG	NM_002746	0.0	1787	141
		Reverse	GCAGAGACTGTAGGTAGTTTCGG				
ERK2 (MAPK2)	MAPK/ERK	Forward	ACACCAACCTCTCGTACATCGG	NM_002745	0.0	5881	131
		Reverse	TGGCAGTAGGTCTGGTGCTCAA				
DUSP6	MAPK/ERK	Forward Reverse	CTCGGATCACTGGAGCCAAAAC GTCACAGTGACTGAGCGGCTAA	NM_001946	0.0	3627	87
TNF-α (Pro-inflammatory cytokine)	Inflammation	Forward Reverse	CTCTTCTGCCTGCTGCACTTTG ATGGGCTACAGGCTTGTCACTC	NM_000594	0.0	2449	146
PAR-1 (Protease-Activated Receptor-1)	PAR Signaling	Forward Reverse	GCTGTCCTACTGCTTGGAAGAC CTGCATCAGCACATACTCCTCC	NM_022002	0.0	2745	169
PAR-2 (Protease-Activated Receptor-2)	PAR Signaling	Forward Reverse	CTCCTCTCTGTCATCTGGTTCC TGCACACTGAGGCAGGTCATGA	NM_005242	0.0	2861	142
GAPDH (Housekeeping control)	Normalization	Forward Reverse	GTCTCCTCTGACTTCAACAGCG ACCACCCTGTTGCTGTAGCCAA	NM_002046	0.0	2374	123

## Data Availability

The datasets generated and/or analyzed during the current study are not publicly available but are accessible from the corresponding author upon reasonable request. Interested researchers may contact the corresponding author for data access inquiries, subject to compliance with any applicable privacy or confidentiality obligations.

## References

[1] Hossain M.S., Karuniawati H., Jairoun A.A., Urbi Z., Ooi J., John A., Lim Y.C., Kibria K.M.K., Mohiuddin A.K.M., Ming L.C. (2022). Colorectal Cancer: A Review of Carcinogenesis, Global Epidemiology, Current Challenges, Risk Factors, Preventive and Treatment Strategies. Cancers.

[2] Aleksandrova K., Nimptsch K., Pischon T. (2013). Influence of Obesity and Related Metabolic Alterations on Colorectal Cancer Risk. Curr. Nutr. Rep..

[3] Wu H., Zhang J., Zhou B. (2021). Metabolic syndrome and colorectal adenoma risk: A systematic review and meta-analysis. Clin. Res. Hepatol. Gastroenterol..

[4] He X., Lan H., Jin K., Liu F. (2023). Cholesterol in colorectal cancer: An essential but tumorigenic precursor?. Front. Oncol..

[5] Liu W., Dong S., Hao F., Gao Y., Wei Q. (2025). Lipid metabolic reprogramming in colorectal cancer: Mechanisms and therapeutic strategies. Front. Immunol..

[6] Istvan E.S., Deisenhofer J. (2001). Structural mechanism for statin inhibition of HMG-CoA reductase. Science.

[7] Morofuji Y., Nakagawa S., Ujifuku K., Fujimoto T., Otsuka K., Niwa M., Tsutsumi K. (2022). Beyond Lipid-Lowering: Effects of Statins on Cardiovascular and Cerebrovascular Diseases and Cancer. Pharmaceuticals.

[8] Juarez D., Fruman D.A. (2021). Targeting the Mevalonate Pathway in Cancer. Trends Cancer.

[9] Lagunas-Rangel F.A., Jonsson J., Jackevica L., Fredriksson R., Dambrova M., Schiöth H.B. (2025). Statins regulate kinase signaling by causing changes in phosphorylation, rather than through changes in gene expression or direct inhibition: Evidence in colorectal cancer. Front. Pharmacol..

[10] Kumar N., Mandal C.C. (2021). Cholesterol-Lowering Drugs on Akt Signaling for Prevention of Tumorigenesis. Front. Genet..

[11] Tripathi S., Gupta E., Galande S. (2024). Statins as anti-tumor agents: A paradigm for repurposed drugs. Cancer Rep..

[12] Wood W.G., Igbavboa U., Muller W.E., Eckert G.P. (2013). Statins, Bcl-2, and apoptosis: Cell death or cell protection?. Mol. Neurobiol..

[13] Sleiman J., Khouri C., Lee U.J., Dimachkie R., Abureesh O., Njeim R., Deeb L. (2025). Chemopreventive Effects of Statins and Aspirin on Colorectal Cancer Among Patients with Inflammatory Bowel Disease: Evidence of a Synergistic Association from a Large Cohort. Clin. Exp. Gastroenterol..

[14] Abbaszadeh M., Bayrami F., Zandifar S., Hemmati H.R., Valadkhani A., Peimanfar A. (2025). Statins and the risk of colorectal cancer; a systematic review and meta-analysis of cohort and case-control studies. Immunopathol. Persa.

[15] Burgos-Molina A.M., Téllez Santana T., Redondo M., Bravo Romero M.J. (2024). The Crucial Role of Inflammation and the Immune System in Colorectal Cancer Carcinogenesis: A Comprehensive Perspective. Int. J. Mol. Sci..

[16] Luo C., Zhang H. (2017). The Role of Proinflammatory Pathways in the Pathogenesis of Colitis-Associated Colorectal Cancer. Mediat. Inflamm..

[17] Li Q., von Ehrlich-Treuenstätt V., Schardey J., Wirth U., Zimmermann P., Andrassy J., Bazhin A.V., Werner J., Kühn F. (2023). Gut Barrier Dysfunction and Bacterial Lipopolysaccharides in Colorectal Cancer. J. Gastrointest. Surg..

[18] Wei J., Zhang Y., Li H., Wang F., Yao S. (2023). Toll-like receptor 4: A potential therapeutic target for multiple human diseases. Biomed. Pharmacother..

[19] Wu Y., Zhou B.P. (2010). TNF-α/NF-κB/Snail pathway in cancer cell migration and invasion. Br. J. Cancer.

[20] Patnaik R., Varghese R.L., Banerjee Y. (2025). Selective Modulation of PAR-2-Driven Inflammatory Pathways by Oleocanthal: Attenuation of TNF-α and Calcium Dysregulation in Colorectal Cancer Models. Int. J. Mol. Sci..

[21] Ayoub M.A., Pin J.P. (2013). Interaction of Protease-Activated Receptor 2 with G Proteins and β-Arrestin 1 Studied by Bioluminescence Resonance Energy Transfer. Front. Endocrinol..

[22] Kawaguchi M., Yamamoto K., Kataoka H., Izumi A., Yamashita F., Kiwaki T., Nishida T., Camerer E., Fukushima T. (2020). Protease-activated receptor-2 accelerates intestinal tumor formation through activation of nuclear factor-κB signaling and tumor angiogenesis in Apc(Min/+) mice. Cancer Sci..

[23] Shah H., Hill T.A., Lim J., Fairlie D.P. (2023). Protease-activated receptor 2 attenuates doxorubicin-induced apoptosis in colon cancer cells. J. Cell Commun. Signal..

[24] Versteeg H.H., Schaffner F., Kerver M., Ellies L.G., Andrade-Gordon P., Mueller B.M., Ruf W. (2008). Protease-activated receptor (PAR) 2, but not PAR1, signaling promotes the development of mammary adenocarcinoma in polyoma middle T mice. Cancer Res..

[25] Nasri I., Bonnet D., Zwarycz B., d’Aldebert E., Khou S., Mezghani-Jarraya R., Quaranta M., Rolland C., Bonnart C., Mas E. (2016). PAR2-dependent activation of GSK3β regulates the survival of colon stem/progenitor cells. Am. J. Physiol. Gastrointest. Liver Physiol..

[26] Patnaik R., Varghese R.L., Khan S., Huda B., Bhurka F., Amiri L., Banerjee Y. (2025). Targeting PAR-2-driven inflammatory pathways in colorectal cancer: Mechanistic insights from atorvastatin and rosuvastatin treatment in cell line models. Transl. Cancer Res..

[27] Pokharel S.M., Chiok K., Shil N.K., Mohanty I., Bose S. (2021). Tumor Necrosis Factor-alpha utilizes MAPK/NFκB pathways to induce cholesterol-25 hydroxylase for amplifying pro-inflammatory response via 25-hydroxycholesterol-integrin-FAK pathway. PLoS ONE.

[28] Guo D., Zhou H., Wu Y., Zhou F., Xu G., Wen H., Zhang X. (2011). Involvement of ERK1/2/NF-κB signal transduction pathway in TF/FVIIa/PAR2-induced proliferation and migration of colon cancer cell SW620. Tumour Biol..

[29] Satow R., Hirano T., Batori R., Nakamura T., Murayama Y., Fukami K. (2014). Phospholipase Cδ1 induces E-cadherin expression and suppresses malignancy in colorectal cancer cells. Proc. Natl. Acad. Sci. USA.

[30] Climent E., Benaiges D., Pedro-Botet J. (2021). Hydrophilic or Lipophilic Statins?. Front. Cardiovasc. Med..

[31] Hirota T., Fujita Y., Ieiri I. (2020). An updated review of pharmacokinetic drug interactions and pharmacogenetics of statins. Expert. Opin. Drug Metab. Toxicol..

[32] Cohen E., Ophir I., Shaul Y.B. (1999). Induced differentiation in HT29, a human colon adenocarcinoma cell line. J. Cell Sci..

[33] Fernando E.H., Gordon M.H., Beck P.L., MacNaughton W.K. (2018). Inhibition of Intestinal Epithelial Wound Healing through Protease-Activated Receptor-2 Activation in Caco2 Cells. J. Pharmacol. Exp. Ther..

[34] Patnaik R., Varghese R., Al-Kabani A., Jannati S., Banerjee Y. (2025). Curcumin Inhibits Protease Activated Receptor 2-Induced ERK Phosphorylation Calcium Mobilization and Anti-Apoptotic Signaling in Inflammation-Driven Colorectal Cancer Cells. Cells.

[35] Khan S., Huda B., Bhurka F., Patnaik R., Banerjee Y. (2025). Molecular and Immunomodulatory Mechanisms of Statins in Inflammation and Cancer Therapeutics with Emphasis on the NF-κB, NLRP3 Inflammasome, and Cytokine Regulatory Axes. Int. J. Mol. Sci..

[36] Kang S.H., Kim G.O., Kim B.Y., Son E.J., Do J.Y. (2023). Correlation between Statin Solubility and Mortality in Patients on Chronic Hemodialysis. Diagnostics.

[37] Lennernäs H. (2003). Clinical pharmacokinetics of atorvastatin. Clin. Pharmacokinet..

[38] McKenney J.M. (2005). Efficacy and safety of rosuvastatin in treatment of dyslipidemia. Am. J. Health Syst. Pharm..

[39] Schachter M. (2005). Chemical, pharmacokinetic and pharmacodynamic properties of statins: An update. Fundam. Clin. Pharmacol..

[40] Ahmadi M., Amiri S., Pecic S., Machaj F., Rosik J., Łos M.J., Alizadeh J., Mahdian R., da Silva Rosa S.C., Schaafsma D. (2020). Pleiotropic effects of statins: A focus on cancer. Biochim. Biophys. Acta Mol. Basis. Dis..

[41] Ma Y., He L., Zhao X., Li W., Lv X., Zhang X., Peng J., Yang L., Xu Q., Wang H. (2021). Protease activated receptor 2 signaling promotes self-renewal and metastasis in colorectal cancer through β-catenin and periostin. Cancer Lett..

[42] Li H., Zhu H., Xu C.J., Yuan J. (1998). Cleavage of BID by caspase 8 mediates the mitochondrial damage in the Fas pathway of apoptosis. Cell.

[43] Walters J., Pop C., Scott F.L., Drag M., Swartz P., Mattos C., Salvesen G.S., Clark A.C. (2009). A constitutively active and uninhibitable caspase-3 zymogen efficiently induces apoptosis. Biochem. J..

[44] Ponder K.G., Boise L.H. (2019). The prodomain of caspase-3 regulates its own removal and caspase activation. Cell Death Discov..

[45] Meyer U., Rönnpagel V., Grammbauer S., von Lucadou M., Rauch-Kröhnert U., Schwedhelm E., Dombrowski F., Ritter C., Rauch B.H. (2025). Significance of FXa and its receptor PAR2 for the growth of colon cancer cells in vitro and in vivo. Front. Oncol..

[46] Jang H.J., Hong E.M., Park S.W., Byun H.W., Koh D.H., Choi M.H., Kae S.H., Lee J. (2016). Statin induces apoptosis of human colon cancer cells and downregulation of insulin-like growth factor 1 receptor via proapoptotic ERK activation. Oncol. Lett..

[47] Lee W., Belkhiri A., Lockhart A.C., Merchant N., Glaeser H., Harris E.I., Washington M.K., Brunt E.M., Zaika A., Kim R.B. (2008). Overexpression of OATP1B3 confers apoptotic resistance in colon cancer. Cancer Res..

[48] Thakkar N., Lockhart A.C., Lee W. (2015). Role of Organic Anion-Transporting Polypeptides (OATPs) in Cancer Therapy. Aaps J..

[49] Elsby R., Martin P., Surry D., Sharma P., Fenner K. (2016). Solitary Inhibition of the Breast Cancer Resistance Protein Efflux Transporter Results in a Clinically Significant Drug-Drug Interaction with Rosuvastatin by Causing up to a 2-Fold Increase in Statin Exposure. Drug Metab. Dispos. Biol. Fate Chem..

[50] Rosenson R.S. (2003). Rosuvastatin: A new inhibitor of HMG CoA reductase for the treatment of dyslipidemia. Expert Rev. Cardiovasc. Ther..

[51] Johnson M., Patel D., Matheny C., Ho M., Chen L., Ellens H. (2017). Inhibition of Intestinal OATP2B1 by the Calcium Receptor Antagonist Ronacaleret Results in a Significant Drug-Drug Interaction by Causing a 2-Fold Decrease in Exposure of Rosuvastatin. Drug Metab. Dispos. Biol. Fate Chem..

[52] Knebel W., Gastonguay M.R., Malhotra B., El-Tahtawy A., Jen F., Gandelman K. (2013). Population pharmacokinetics of atorvastatin and its active metabolites in children and adolescents with heterozygous familial hypercholesterolemia: Selective use of informative prior distributions from adults. J. Clin. Pharmacol..

[53] European Medicines Agency (EMA) Lipitor: Article 30 Referral Annex III. https://www.ema.europa.eu/en/documents/referral/lipitor-article-30-referral-annex-iii_en.pdf.

[54] U.S. Food and Drug Administration (FDA) (2009). Lipitor (Atorvastatin Calcium) Tablets. For Oral Use: Prescribing Information (Label). https://www.accessdata.fda.gov/drugsatfda_docs/label/2009/020702s057lbl.pdf.

[55] Zhu Y., Casey P.J., Kumar A.P., Pervaiz S. (2013). Deciphering the signaling networks underlying simvastatin-induced apoptosis in human cancer cells: Evidence for non-canonical activation of RhoA and Rac1 GTPases. Cell Death Dis..

[56] Nam G.H., Kwon M., Jung H., Ko E., Kim S.A., Choi Y., Song S.J., Kim S., Lee Y., Kim G.B. (2021). Statin-mediated inhibition of RAS prenylation activates ER stress to enhance the immunogenicity of KRAS mutant cancer. J. Immunother. Cancer.

[57] Patil P.S., Saklani A., Kumar N.A.N., De’Souza A., Krishnatry R., Khanvilkar S., Kazi M., Engineer R., Ostwal V., Ramaswamy A. (2025). A randomized phase II/III trial of rosuvastatin with neoadjuvant chemo-radiation in patients with locally advanced rectal cancer. Front. Oncol..

[58] Alizadeh J., Zeki A.A., Mirzaei N., Tewary S., Rezaei Moghadam A., Glogowska A., Nagakannan P., Eftekharpour E., Wiechec E., Gordon J.W. (2017). Mevalonate Cascade Inhibition by Simvastatin Induces the Intrinsic Apoptosis Pathway via Depletion of Isoprenoids in Tumor Cells. Sci. Rep..

[59] Sun J., Halfvarson J., Bergman D., Ebrahimi F., Roelstraete B., Lochhead P., Song M., Olén O., Ludvigsson J.F. (2023). Statin use and risk of colorectal cancer in patients with inflammatory bowel disease. EClinicalMedicine.

[60] Li Y., He X., Ding Y., Chen H., Sun L. (2019). Statin uses and mortality in colorectal cancer patients: An updated systematic review and meta-analysis. Cancer Med..

[61] Tsubaki M., Takeda T., Matsuda T., Kishimoto K., Takefuji H., Taniwaki Y., Ueda M., Hoshida T., Tanabe K., Nishida S. (2023). Statins enhances antitumor effect of oxaliplatin in KRAS-mutated colorectal cancer cells and inhibits oxaliplatin-induced neuropathy. Cancer Cell Int..

[62] Yuan T., Wu R., Wang W., Liu Y., Kong W., Yang B., He Q., Zhu H. (2023). Synergistic antitumor activity of regorafenib and rosuvastatin in colorectal cancer. Front. Pharmacol..

[63] Hoffmeister M., Jansen L., Rudolph A., Toth C., Kloor M., Roth W., Bläker H., Chang-Claude J., Brenner H. (2015). Statin use and survival after colo-rectal cancer: The importance of com-prehensive con-founder adjustment. J. Natl. Cancer Inst..

[64] Bertagnolli M.M., Hsu M., Hawk E.T., Eagle C.J., Zauber A.G. (2010). Statin use and colorectal adenoma risk: Results from the adenoma prevention with celecoxib trial. Cancer Prev. Res..

[65] Lytras T., Nikolopoulos G., Bonovas S. (2014). Statins and the risk of colorectal cancer: An updated systematic review and meta-analysis of 40 studies. World J. Gastroenterol..

[66] Li L., Cui N., Hao T., Zou J., Jiao W., Yi K., Yu W. (2021). Statins use and the prognosis of colorec-tal cancer: A meta-analysis. Clin. Res. Hepatol. Gastroenterol..

[67] Janakiram N.B., Mohammed A., Bryant T., Zhang Y., Brewer M., Duff A., Biddick L., Singh A., Lightfoot S., Steele V.E. (2016). Potentiating NK cell activity by combination of Rosuvastatin and Difluoromethylornithine for effective chemopreventive efficacy against Colon Cancer. Sci. Rep..

[68] Iablokov V., Hirota C.L., Peplowski M.A., Ramachandran R., Mihara K., Hollenberg M.D., MacNaughton W.K. (2014). Proteinase-activated receptor 2 (PAR2) decreases apoptosis in colonic epithelial cells. J. Biol. Chem..

[69] Quan Q., Zhong F., Wang X., Chen K., Guo L. (2019). PAR2 Inhibition Enhanced the Sensitivity of Colorectal Cancer Cells to 5-FU and Reduced EMT Signaling. Oncol. Res..

[70] Darmoul D., Gratio V., Devaud H., Laburthe M. (2004). Protease-activated receptor 2 in colon cancer: Trypsin-induced MAPK phosphorylation and cell proliferation are mediated by epidermal growth factor receptor transactivation. J. Biol. Chem..

[71] Kawabata A., Matsunami M., Tsutsumi M., Ishiki T., Fukushima O., Sekiguchi F., Kawao N., Minami T., Kanke T., Saito N. (2006). Suppression of pancreatitis-related allodynia/hyperalgesia by proteinase-activated receptor-2 in mice. Br. J. Pharmacol..

[72] Ting A.T., Bertrand M.J.M. (2016). More to Life than NF-κB in TNFR1 Signaling. Trends Immunol..

[73] Xu J. (2025). The role of tumor necrosis factor receptor superfamily in cancer: Insights into oncogenesis, progression, and therapeutic strategies. NPJ Precis. Oncol..

[74] Dang Y., Zhang Y., Wang Z. (2025). The role of statins in the regulation of breast and colorectal cancer and future directions. Front. Pharmacol..

[75] Goc A., Kochuparambil S.T., Al-Husein B., Al-Azayzih A., Mohammad S., Somanath P.R. (2012). Simultaneous modulation of the intrinsic and extrinsic pathways by simvastatin in mediating prostate cancer cell apoptosis. BMC Cancer.

[76] Spampanato C., De Maria S., Sarnataro M., Giordano E., Zanfardino M., Baiano S., Cartenì M., Morelli F. (2012). Simvastatin inhibits cancer cell growth by inducing apoptosis correlated to activation of Bax and down-regulation of BCL-2 gene expression. Int. J. Oncol..

[77] Ahn K.S., Sethi G., Aggarwal B.B. (2007). Simvastatin potentiates TNF-alpha-induced apoptosis through the down-regulation of NF-kappaB-dependent antiapoptotic gene products: Role of IkappaBalpha kinase and TGF-beta-activated kinase-1. J. Immunol..

[78] Chang H.L., Chen C.Y., Hsu Y.F., Kuo W.S., Ou G., Chiu P.T., Huang Y.H., Hsu M.J. (2013). Simvastatin induced HCT116 colorectal cancer cell apoptosis through p38MAPK-p53-survivin signaling cascade. Biochim. Biophys. Acta.

[79] Fong C.W. (2014). Statins in therapy: Understanding their hydrophilicity, lipophilicity, binding to 3-hydroxy-3-methylglutaryl-CoA reductase, ability to cross the blood brain barrier and metabolic stability based on electrostatic molecular orbital studies. Eur. J. Med. Chem..

